# Die Erforschung der Dynamik der Corona-Pandemie in Deutschland: Survey-Konzepte und eine exemplarische Umsetzung mit dem Sozio-oekonomischen Panel (SOEP)

**DOI:** 10.1007/s11943-021-00296-x

**Published:** 2021-12-09

**Authors:** Ulrich Rendtel, Stefan Liebig, Reinhard Meister, Gert G. Wagner, Sabine Zinn

**Affiliations:** 1 Freie Universität Berlin, Berlin, Deutschland; 2 Sozio-oekonomisches Panel (SOEP), Berlin, Deutschland; 3 Berliner Hochschule für Technik (BHT), Berlin, Deutschland; 4 Max PIanck Institut für Bildungsforschung, Berlin, Deutschland; 5 Humboldt Universität, Berlin, Deutschland

**Keywords:** Corona Pandemie, Infektions-Survey, Sozio-oekonomisches Panel (SOEP), WHO, Dunkelziffer, Inzidenz-Raten, Corona pandemic, Infection Survey, Socio-economic Panel (SOEP), WHO, Hidden cases, Incidence rates, I12, I18

## Abstract

Die Weltgesundheitsorganisation (WHO) hat im Frühjahr 2020 Richtlinien für Bevölkerungsstichproben veröffentlicht, die Basisdaten für gesundheitspolitische Entscheidungen im Pandemiefall liefern können. Diese Richtlinien umzusetzen ist keineswegs trivial. In diesem Beitrag schildern wir die Herausforderungen einer entsprechenden statistischen Erfassung der Corona Pandemie. Hierbei gehen wir im ersten Teil auf die Erfassung der Dunkelziffer bei der Meldung von Corona Infektionen, die Messung von Krankheitsverläufen im außerklinischen Bereich, die Messung von Risikomerkmalen sowie die Erfassung von zeitlichen und regionalen Veränderungen der Pandemie-Intensität ein. Wir diskutieren verschiedene Möglichkeiten, aber auch praktische Grenzen der Survey-Statistik, den vielfältigen Herausforderungen durch eine geeignete Anlage der Stichprobe und des Survey-Designs zu begegnen. Ein zentraler Punkt ist die schwierige Koppelung medizinischer Tests mit bevölkerungsrepräsentativen Umfragen, wobei bei einer personalisierten Rückmeldung der Testergebnisse das Statistik-Geheimnis eine besondere Herausforderung darstellt.

Im zweiten Teil berichten wir wie eine der großen Wiederholungsbefragungen in Deutschland, das Sozio-oekonomische Panel (SOEP), für eine WHO-konforme Covid-19-Erhebung genutzt wird, die im Rahmen einer Kooperation des Robert-Koch-Instituts (RKI) mit dem SOEP als „RKI-SOEP Stichprobe“ im September 2020 gestartet wurde. Erste Ergebnisse zum Rücklauf dieser Studie, die ab Oktober 2021 mit einer zweiten Erhebungswelle bei denselben Personen fortgesetzt werden wird, werden vorgestellt. Es zeigt sich, dass knapp fünf Prozent der bereits in der Vergangenheit erfolgreich Befragten aufgrund der Anfrage zwei Tests zu machen die weitere Teilnahme an der SOEP-Studie verweigern. Berücksichtigt man alle in der Studie erhobenen Informationen (IgG-Antikörper-Tests, PCR-Tests und Fragebögen) ergibt eine erste Schätzung, dass sich bis November 2020 nur etwa zwei Prozent der in Privathaushalten lebenden Erwachsenen in Deutschland mit SARS-CoV‑2 infiziert hatten. Damit war die Zahl der Infektionen etwa doppelt so hoch wie die offiziell gemeldeten Infektionszahlen.

## Abstract (en)

In Spring 2020 the World Health Organization (WHO) has published guidelines for population surveys which can deliver baseline data for health policy decisions in pandemic situations. The realization of the guidelines is by no means trivial. In this essay we describe the challenges of a statistical assessment of the Corona Pandemic. In the first part we treat the extent of hidden cases in the registration of Corona infections, the measurement of infection histories outside the clinical sector, the measurement of risk factors and finally the measurement of temporal and regional changes of the intensity of the pandemic. We discuss several possibilities but also limitations of surveys statistics to cope with the manifold challenges by a proper survey concept and design of a sample. A central issue is the difficult link of medical tests with representative populations surveys where confidentiality rules in the case of individual feedback of test results pose a special challenge.

In the second part we report how one of the large German panel surveys, the SOcio-Economic Panel (SOEP), is used for a Covid-19 survey which is in line with the WHO guidelines. This survey was started in September 2020 in the framework of a cooperation of the Robert-Koch-Institut (RKI) with the SOEP as the “RKI-SOEP Stichprobe”. First results on the response rate of the survey which is prolongated in October 2021 are reported. It turned out that 5 percent of the previously cooperative interviewees refuse the further participation in the SOEP because of the invitation to the participate in the medical testing. On the basis of all available survey information (IgG antibody test, PCR test and questionnaire) a first estimate reveals that until November 2020 only two percent of the German adults in private households have been infected with SARS-CoV‑2. This number of infections is about twice as high as the officially reported infection cases.

## Einleitung

Die durch das Coronavirus (SARS-CoV‑2) ausgelöste Pandemie stellt weltweit ein akutes Gesundheitsproblem dar. Bis Oktober 2021 gab es 248 Mio. bestätigte Infizierte und 5 Mio. am oder mit dem Corona-Virus Verstorbene (Stand 17. Oktober 2021). Die Pandemie hat im Verbund mit teilweise drastischen Eindämmungsmaßnahmen zu gravierenden ökonomischen und sozialen Konsequenzen geführt, deren mittel- und langfristige Wirkungen noch nicht abzusehen sind.

In Deutschland basierten die Maßnahmen zur Eindämmung der Coronavirus-Disease-2019 (COVID‑19) nach kurzer Zeit der Orientierung (in der das Konzept der Verdopplungszeit anfänglich eine große Rolle spielte) neben der Auslastung der Intensivstationen auf der Anzahl der in 7 Tagen gemeldeten Neuinfektionen pro 100 Tsd. Einwohner, der sogenannten 7‑Tages-Inzidenz. Diese wird in Deutschland regional differenziert pro Landkreis täglich vom Robert-Koch-Institut (RKI) bekannt gegeben. Einige Monate lang galt die Marke von 50 als Grenze für Lockerungen beziehungsweise Verschärfungen von Eindämmungsmaßnahmen. Dieser Inzidenzwert ist im Juli 2020 im Infektionsschutzgesetz (§ 28a, Absatz 3) als Grenzwert für „umfassende Schutzmaßnahmen“ fixiert worden. Daneben wurden für Deutschland auch Hochrisikogebiete definiert, in denen der Inzidenzwert über dem Wert von 200 liegt.

**Figure 1:**
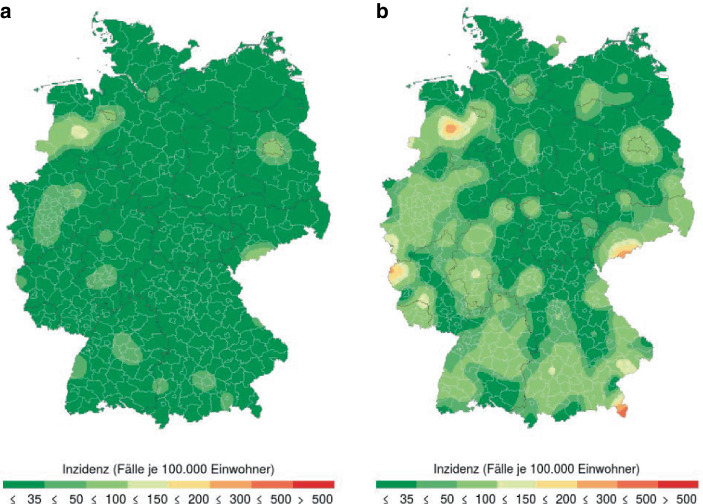

Insbesondere die Inzidenzzahlen, die auf gemeldeten Infektionen beruhen, als auch die Verwendung bestimmter Schwellenwerte sind nicht unumstritten. Denn nicht die Anzahl der Infizierten gilt als eigentlicher Zielwert, sondern die Inzidenz-Zielwerte sind Hilfsindikatoren für die Fähigkeit der Gesundheitsämter alle Kontaktpersonen von Infizierten ermitteln und isolieren zu können und so das Infektionsgeschehen kontrollieren zu können^1^^,^^2^. Allerdings hat sich der statistisch gemessene Inzidenzwert als kein guter Indikator für die Beherrschung der Pandemie durch die Gesundheitsämter herausgestellt^3^. So lag die 7 Tages-Inzidenz in Deutschland vor dem Beginn der zweiten Welle ab Oktober 2020 weit unter 50, nämlich bei 15. Trotz dieses angeblich beherrschbaren Infektionsniveaus hat dies die Ausbreitung der zweiten Corona-Infektionswelle nicht verhindert. Während in der Woche vom 02.10.2020 bis zum 08.10.2020 noch deutlich getrennte Infektionsgebiete zu erkennen waren, hatten sich diese schon 11 Tage später deutlich vergrößert und begonnen sich über ganz Deutschland auszubreiten, vgl. Abb. 1,^4^.

**Figure 2:**
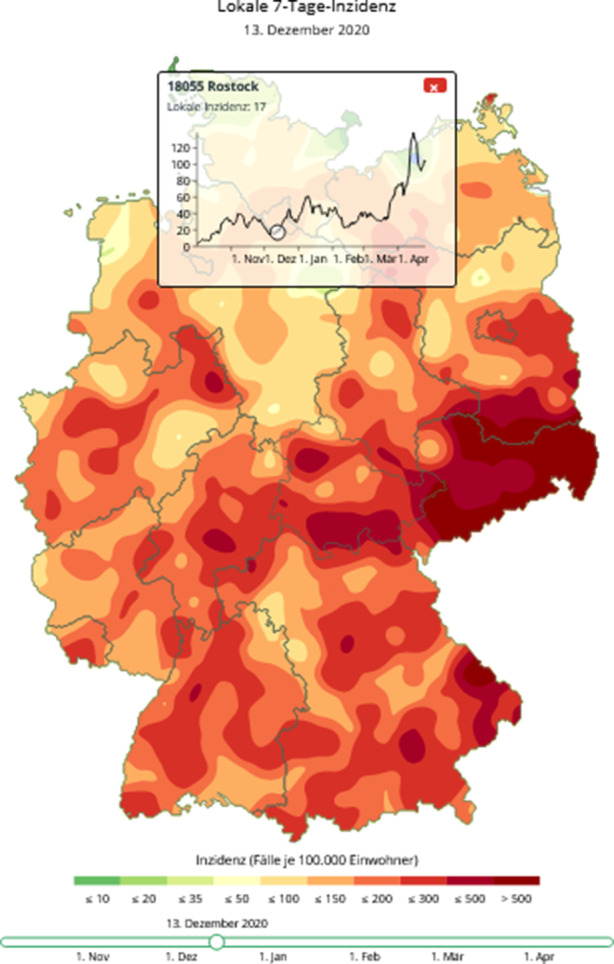

Statt dem Hilfsindikator Inzidenz zu vertrauen, wurde in Rostock die erst seit dem Frühjahr 2021 bundesweit massiv angewendete freiwillige und unentgeltliche Testung der Bevölkerung frühzeitig eingesetzt. Diese Präventiv-Testung^5^ zeigte eine messbare Wirkung: Rostock blieb fast während der gesamten zweiten Corona-Welle ein „Cold-Spot“^6^. Dies wird auch in Abb. 2 deutlich, wo Rostock und Umland kurz vor dem Höhepunkt der zweiten Welle als eine der wenigen Inseln in einer Umgebung von Hotspots auftaucht.

**Figure 3:**
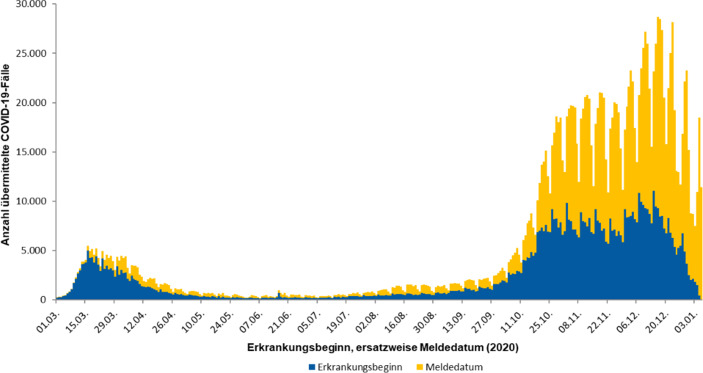

Ein grundsätzliches Problem besteht darin, dass die gemeldeten Infektionszahlen nicht mit der Anzahl der Infizierten identisch sind. Die offiziellen Infektionszahlen werden über die Anzahl der positiv auf das Corona Virus getesteten Personen bestimmt – und die Testhäufigkeit variiert mit der Zeit. Zusätzlich zeigt sich als Resultat des wöchentlichen Arbeitsrhythmus der Gesundheitsämter ein ausgeprägtes wochentägliches Saisonalitätsmuster der Infektionszahlen, das keinesfalls mit einem wöchentlichen Erkrankungsschema zu verwechseln ist, vgl. Abb. 3.^7^

Getestet wurden zu Anfang der Pandemie Personen mit Krankheitssymptomen und deren Kontaktpersonen. Im weiteren Verlauf der Pandemie wurde der Kreis der zu testenden Personen auf die Mitarbeiter in Krankenhäusern und Alters- und Pflegeheimen ausgeweitet. Dadurch stieg zunächst die Anzahl der Testungen und damit auch die Zahl der entdeckten Corona-Infektionen. Diese schwankende Testintensität zieht ihrerseits schwankende Inzidenzzahlen nach sich, so dass steigende Inzidenzen auch das Resultat vermehrten Testens sein können.

**Figure 4:**
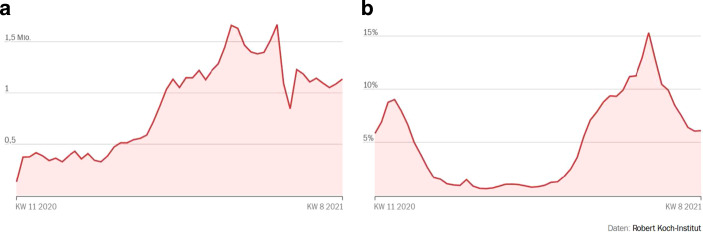

**Figure 5:**
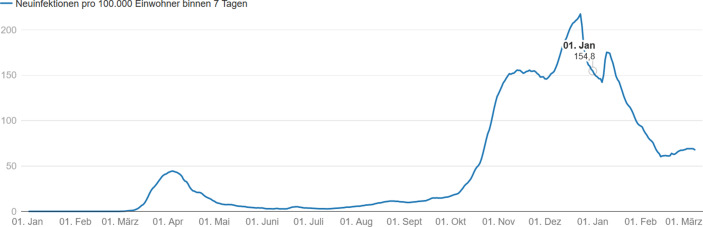

Besonders stark schwankten die Testungen um den Jahreswechsel 2020/2021, vgl. Abb. 4a. So halbierte sich die Anzahl Tests von 1,67 Mio. in der Woche vom 14. Dez. bis 20. Dez. 2020 auf 0,845 Mio. in der Woche vom 28. Dez. 2020 bis 3. Jan. 2021. Die Positivrate der Tests stieg dabei von 11,3 auf 15,4 % an. Dieser Wert markiert zugleich den Maximalwert während der zweiten Corona-Welle, vgl. Abb. 4b. Das Resultat dieser gegenläufigen Bewegungen ist die in Abb. 5 dargestellte Entwicklung der Inzidenzwerte in Deutschland. Sehr wahrscheinlich gibt somit der U‑förmige Verlauf der Inzidenzkurve um die Jahreswende 2020/2021 die Entwicklung der Corona-Epidemie nicht korrekt wieder. Diese dürfte etwa zum Jahreswechsel ihren Gipfelpunkt erreicht haben.

Ein weiteres Beispiel für irreführende Befunde auf Basis gemeldeter Inzidenzzahlen basiert auf der zeitlich wechselnden Testung von Kindern. Tab. 1 vergleicht die Inzidenzwerte des RKI für verschiedene Altersgruppen^8^. Während in der Kalenderwoche 50 (2020) die meisten Schulen und Kitas für das Gros der Kinder geschlossen waren, läuft in der Kalenderwoche 15 (2021) der Schul- und Kitabetrieb noch in den meisten Fällen^9^. In beiden Kalenderwochen liegt der Inzidenzwert in der Altersgruppe 20 bis 24 auf dem gleichen relativ hohen Infektionsniveau (224 in Kalenderwoche 50 bzw. 249 in Kalenderwoche 15). Diese Altersgruppe ist nicht von der Öffnung bzw. Schließung der Schulen betroffen. Allerdings haben sich die Inzidenzwerte für die Gruppe der Schüler und Kitabesucher in Woche 15 gegenüber der Woche 50 ungefähr verdoppelt und liegen jetzt in der Größenordnung der 20- bis 25-Jährigen. Ganz offensichtlich ist dieser Anstieg das Resultat einer verstärkten Testung der Jugendlichen in den Schulen und Kitas und nicht Resultat einer verstärkten Erkrankung von Kindern und Jugendlichen^10^.

**Table 1:** 

Altersgruppe	Kalenderwoche 50 (2020)	Kalenderwoche 15 (2021)
	7.–13 Dez. 2020	12.–18. April 2021
20–24	224	249
15–19	154	271
10–14	114	205
5–9	67	185

Es gehört zu den Eigenschaften des SARS-CoV‑2-Virus, dass viele Infizierte gar keine manifesten Krankheitssymptome entwickeln und trotzdem das Virus weiterverbreiten. Deswegen ist für die Ermittlung des Infektionsgeschehens in der Bevölkerung eine (auf einer Zufallsstichprobe basierende) Erfassung der gesamten Bevölkerung notwendig, da nur so die Dunkelziffer der Infektionen ermittelt werden und das Ansteckungsgeschehen valide und unverzerrt für die gesamte in Deutschland lebende Wohnbevölkerung empirisch bestimmt werden kann. Dabei ist es wünschenswert, das Infektionsgeschehen demographisch und sozial zu differenzieren, um die Bevölkerungsgruppen zu identifizieren, die am stärksten betroffen bzw. gefährdet sind. Dies wird auch in der entsprechenden Richtlinie^11^ der WHO (2020) betont, die zu Beginn der Pandemie im Frühjahr 2020 veröffentlicht wurde, vgl. auch Abbott (2020).

Es gehört zu den irritierenden Befunden, dass Deutschland auch nach einem Jahr Pandemie über kein etabliertes statistisches Instrumentarium zur aktuellen Messung der Corona-Infektionen unter Berücksichtigung der Dunkelziffer verfügt. Für England^12^ wird beispielsweise vom Nationalen Statistikamt (ONS) seit Mai 2020 eine repräsentative Panelstudie auf Basis des dortigen Annual Population Survey (APS) betrieben, der alle 14 Tage insgesamt 150.000 Personen testet. Diese Studie wird seit Oktober 2020 als Covid-19 Infection Survey (CIS) auf nationalem Level durchgeführt und läuft bei Erstellung dieses Manuskripts (Juni 2021) immer noch^13^. Die Studie schließt alle Haushaltsmitglieder ab einem Alter von 2 Jahren ein. Eine britische Corona Warn-App bietet 2,4 Mio. Nutzern einen PCR-Test an, wann immer sie Corona-Symptome fühlen^14^.

Neben einer realistischen Erfassung der Anzahl der tatsächlichen Corona-Infektionen sind auch Prognosen für den Bedarf an Krankenhausbetten, Intensivplätzen und Beatmungsgeräten wichtige Aufgaben der statistischen Erfassung der Corona Pandemie. Eine zuverlässige, frühzeitige Prognose würde es dem Gesundheitssystem erleichtern, effektive Planung zu betreiben und Ressourcen zu verteilen. Denn letzten Endes ist es die Verhinderung einer Überlastung des Gesundheitssystems, z. B. gemessen über die Anzahl der freien Intensivbetten, mit der Lockdowns begründet wurden und immer noch werden^15^.

Über die Langfristfolgen einer Corona-Infektion, insbesondere über die Dauer der Immunität nach einer überstandenen Infektion, liegen auch nach einem Jahr der Pandemie naturgemäß noch keine belastbaren Befunde vor. Dies gilt auch für die Dauer der Immunität nach einer Impfung gegen das Corona Virus. Diese Situation begründet ein Mandat für eine andauernde Beobachtung von Corona-Infizierten.

Corona-Infektionen werden über Aerosole übertragen. Dies begünstigt Ansteckungen im unmittelbaren persönlichen Umfeld, so dass Corona-Ausbrüche nicht selten in Clustern von mehreren Infizierten auftreten. Von daher sind Betriebe, Alters- und Pflegeheime, Krankenhäuser, Schulen aber eben auch private Haushalte Orte mit hohem Ansteckungsrisiko. Erhebungstechnisch sollten daher Haushalte statt Einzelpersonen auf ihren Infektionsstatus hin untersucht werden.

Zusätzlich sollte das Corona-Ansteckungsrisiko sozial und demographisch differenziert untersucht werden. So hatte man schon zu Beginn der Pandemie Menschen über 70 Jahre und Personen mit Vorerkrankungen als Risikopersonen eingestuft. Jedoch zeigt sich mit fortschreitender Dauer der Pandemie, dass auch jüngere Personen ernstzunehmende Krankheitsverläufe durchmachen, so dass auch hier eine genauere Auswertung der Hintergrundmerkmale sinnvoll ist. Die Rekonstruktion der sozialen Bedingungen des Infektionsgeschehens ist ein zentrales Anliegen der Epidemiologie. Ferner sind auch die ökonomischen und sozialen Folgen der Einschränkung des öffentlichen und privaten Lebens für die Gesellschaft und bestimmte Personengruppen (wie z. B. prekär Beschäftigte oder Familien mit Kindern) zu ermitteln. Die Folgen der Pandemie können sich sehr unterschiedlich auswirken, je nachdem wie stark man direkt oder indirekt von den Corona-Maßnahmen betroffen ist. Die Erforschung dieser Folgewirkungen ist ein zentrales Anliegen der Wirtschafts- und Sozialforschung.

In diesem Artikel beschreiben wir zunächst die Gegenstände und Möglichkeiten einer statistischen Erforschung der Corona-Pandemie und berichten über die bis Frühjahr 2021 erreichten Ziele. Schließlich stellen wir die RKI-SOEP Studie vor, die etliche der geforderten Potenziale zur Analyse der Corona-Pandemie bietet. Diese Studie ist Ende September 2020 zu Beginn der Zweiten Corona-Welle gestartet worden. Eine erste Ideenskizze findet sich in Rendtel et al. (2020). Ein Studienprotokoll ist mittlerweile ebenfalls verfügbar, vgl. Hoebel et al. (2021a). Inzwischen liegen erste Erfahrungen aus der Feldarbeit vor, über die wir hier kurz berichten. Der Aufsatz schließt mit Überlegungen zur Rolle und den Möglichkeiten von wissenschaftlichen Erhebungen für die Kontrolle einer Pandemie.

## Gegenstände einer relevanten statistischen Erforschung der Corona Pandemie

### Dunkelziffern

Für die statistische Beschreibung der Corona-Pandemie ist die Höhe der Dunkelziffer, d. h. die Anzahl der Infizierten, deren Infektion nicht den Gesundheitsämtern gemeldet wurde, von großer Bedeutung. Dabei ist eine genauere Festlegung des Zeitbezugs sinnvoll. Hier kann man zwei Versionen unterscheiden: Die spontane Dunkelziffer sowie die kumulative Dunkelziffer.

Die *spontane Dunkelziffer* misst die Corona-Infektionen, die nicht durch einen Corona-Test (PCR- oder Rapid-Antibody-Test) auf eine akute Infektion aufgedeckt wurden. Das heißt, wird das Ansteckungsrisiko lediglich auf Basis von Infektionen ermittelt, die durch manifeste und anlassbezogene Corona-Tests aufgedeckt werden, führt die spontane Dunkelziffer zu einer Unterschätzung des Ansteckungsrisikos. Die spontane Dunkelziffer kann nur über eine Vollerhebung oder eine zufällige Stichprobe aus der Bevölkerung ermittelt werden, in der alle Personen – ohne Vorbedingung wie Symptome oder einen Kontakt – auf eine akute SARS-Cov‑2 Infektion getestet werden. Als Resultat erhält man die (geschätzte) Summe aller akuten Infektionen zu einem bestimmten Zeitpunkt (bzw. in einem kurzen Zeitraum) in der Bevölkerung. Diese kann mit der Summe aller in diesem Zeitraum über einen PCR-Test positiv getesteten Personen (bei gegeben Testungsregeln) verglichen werden. Dieses Verhältnis ergibt dann den Faktor, mit dem die anlassbezogen aufgedeckten Neuinfektionen multipliziert werden müssen um die tatsächlich Infizierten in der Bevölkerung zu schätzen.

Die *kumulative Dunkelziffer* vergleicht die Anzahl der Personen, die bis zum Messzeitpunkt eine Corona-Infektion durchlaufen haben, mit der kumulativen Anzahl aller bestätigten Corona-Infektionen in der Population. Diese Ziffer ist wichtig für die Bestimmung des Prozentsatzes der Personen, die gegen das Corona-Virus (eine Zeit lang) immun geworden ist. Übersteigt dieser Wert eine gewisse Schwelle, die sogenannte Herdenimmunität, so kann sich das Virus nur noch sehr langsam in der Population weiterverbreiten und die Zahl der täglichen Neuinfektionen sinkt gegen Null. Die Schätzung auf Basis einer Zufallsstichprobe erfolgt durch einen Test auf Antikörper^16^, die sich als Resultat einer durchlaufenen Corona-Infektion gebildet haben.

Obwohl zwischen beiden Dunkelziffern eine enge logische Beziehung steht, sind die konkreten Testinstrumente und ihre Güte sehr verschieden, vgl. Abschn. 4 unten. Auch wächst die kumulative Anzahl der Infektionen kontinuierlich im Verlauf der Pandemie, so dass das Ergebnis vom Zeitpunkt der Messung im Zeitverlauf abhängig ist. Für die spontane Dunkelziffer existiert eine zeitlich stabile Relation nicht per se. Sie kann von verschiedenen Faktoren wie zum Beispiel einer Veränderung der Teststrategien, zum Beispiel dem massenhaften Einsatz von freiwilligen Testungen, dem Auftreten von neuen Virusvarianten mit stärkeren Symptomen oder dem Einsatz von Impfstoffen, die Infektionen verhindern, abhängig sein.

Bis zum September 2020 sind in Deutschland Studien, die auch Nicht-Erkrankte und Nicht-Kontaktpersonen erfassen, nur an Brennpunkten (Hot Spots) der COVID‑19-Erkrankung durchgeführt worden, so in Gangelt („Heinsberg Studie“, vgl. Streeck et al. 2020), in München (vgl. Radon et al. 2020), in Kupferzell im Hohenlohekreis (vgl. Lampert und Santos-Hövener 2020) oder zuletzt in Berlin-Mitte^17^. Das RKI listet Anfang März 2021 allein 18 regionale Studien auf^18^. Die Ergebnisse dieser Studien sind strenggenommen nur für den betrachteten Brennpunkt sowie das abgedeckte Zeitintervall aussagekräftig. Doch die Beschränkung auf die Hotspots hat den Vorteil, dass in der Auswahlgesamtheit das interessierende Merkmal, also eine Corona-Infektion, häufiger als in der Gesamtpopulation vorkommt und somit auch genügend aussagekräftige Fallzahlen von Infizierten in der Stichprobe vorliegen. Das RKI gibt für seine Regionalstudien (abgesehen von Berlin-Mitte) eine monatliche Inzidenz von mindestens 500 Fällen pro 100.000 Einwohnern an.

Neben der Feststellung der Infektionsinzidenz nennt das Studienprotokoll der RKI-Regionalstudien^19^ die Möglichkeit, den Einfluss zahlreicher Expositionsmerkmale auf eine Corona-Infektion zu analysieren. Hierzu werden im Rahmen von Fragebögen (retrospektive) Informationen, beispielsweise zum Reise- und Hygieneverhalten, ermittelt. Mögliche multivariate Zusammenhänge sollen dann auf die Gesamtbevölkerung verallgemeinert werden.

Das Ergebnis des Tests auf Antikörper kann weiterhin mit der retrospektiven Befragung zu früheren Corona-Erkrankungen bzw. positiven Akut-Tests verglichen werden. Dies liefert die Möglichkeit eine kumulative Dunkelziffer zu schätzen. Weiterhin muss bei signifikanter Pandemie-Sterblichkeit eine Korrektur für an Corona verstorbene Personen durchgeführt werden, da dieser Personenkreis für die Tests auf Antikörper ausfällt. Problematisch erweisen sich freilich Fälle, in denen der Test auf Antikörper negativ ausfällt, jedoch in einem zugehörigen Fragebogenteil eine Corona-Infektion angegeben worden ist. Man kann dies als falsche Angabe im Fragebogen, als (statistischen) Fehler des Testverfahrens oder aber auch als ein Abklingen der Immunität nach einer Corona-Infektion ansehen. Momentan ist hier noch wenig gesichertes Wissen vorhanden. Je nachdem wie man diese Fälle wertet, erhält man unterschiedliche Dunkelziffern^20^.

Im November 2020 wurde der Endbericht der sogenannten Corona-Bund Studie vorgelegt (vgl. Ifo Institut/Forsa 2020). Diese liefert auf Basis einer Telefon-Dauerstichprobe erstmalig eine kumulative Dunkelziffer für Deutschland für zwei Zeitpunkte: August 2020 und Oktober/November 2020. Danach wird die Anzahl aller Infizierten im Verhältnis zu den vom RKI bestätigten Fälle im Oktober und November mit einem Multiplikator von 2,2 errechnet, während dieser Multiplikator im August noch bei 1,8 gelegen hatte^21^. Streeck et al. (2020) errechnen bei ihrer Hotspot Studie aus der Frühphase der Corona-Pandemie indes mit einer vier- bis fünffachen Zahl von Corona-Infizierten über der Zahl der bestätigten Infektionen hinaus. Wagner et al. (2021) melden für ihre Hotspot-Studie im Landkreis Tirschenreuth vom Juli 2020 einen durchschnittlichen Dunkelzifferfaktor von 5. Allerdings schwankt dieser Faktor erheblich über die Altersgruppen. Während für Jugendliche über 14 Jahren^22^ ein Dunkelzifferfaktor von 12,2 angegeben wird, ist es in der Bevölkerung 85+ nur noch ein Faktor von 1,7. Dieses Ergebnis verwundert angesichts der hohen Anzahl asymptomatischer Krankheitsverläufe bei Jugendlichen nicht. Insgesamt belegen die bisherigen empirischen Ergebnisse eine hohe regionale (Hotspot vs. Population) und zeitliche Instabilität (Infektionswellen) der Dunkelziffer.

### Die Belastung des Klinischen Bereichs und die Dauer und Intensität von Krankenhausaufenthalten

Das Auftreten von Infektionsclustern und die damit verbundenen Belastungen des Gesundheitssystems, insbesondere die Anzahl der Krankenhausaufenthalte, deren Länge und die Intensität der Behandlung sind von großer Bedeutung für das Verstehen und das Management einer Pandemie. Zu einem rationalen Management gehört auch die Bestimmung von Risikofaktoren, die besonders schutzbedürftige Teile der Bevölkerung charakterisieren. Diese Aspekte waren Grundlage der ersten Projektskizze des RKI-SOEP Covid-19 Panels (Rendtel et al. 2020).

Mittlerweile sind viele weitere Aspekte hinzugekommen, wie Impfraten, Virusmutationen, Impfnebenwirkungen etc.. Dennoch lohnt es sich, den ursprünglichen Ansatz zu betrachten, denn dieser sollte nicht nur die Schätzung der erwarteten Anzahl Betroffener ermöglichen. Darüber hinaus war es das Ziel, Grundlagen für ein Prognosemodell zu erarbeiten, welches flexible, angepasste Reaktionen auf das Infektionsgeschehen wiederkehrender Covid-19 Pandemie-Zustände ermöglicht. Es war bekannt, dass an der Universität Freiburg Aktivitäten zum Einsatz von Mehrstadien-Modellen (Multi-State Models) zur Beschreibung des Krankheitsverlaufs von Covid-19 erkrankten Patienten im Gange waren.

**Figure 6:**
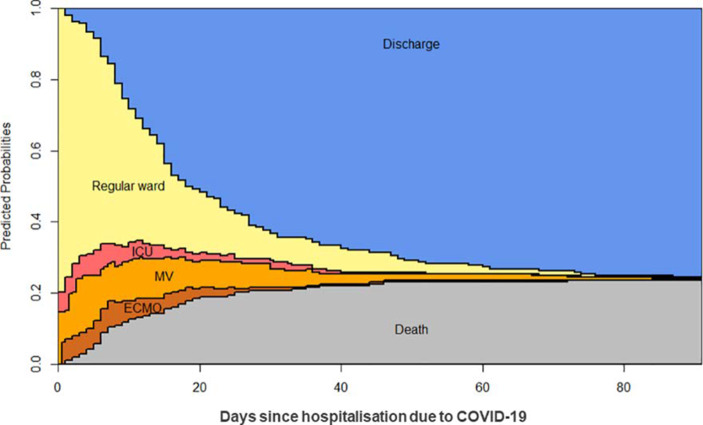

Rieg et al. (2020) haben den Verlauf des Krankenhausaufenthalts für 213 COVID‑19 Patienten untersucht, die zwischen Februar und Mai 2020 in der Freiburger Universitätsklinik eingewiesen wurden. Sie unterscheiden dabei vier Behandlungsstadien: Reguläre Station (Reg. Ward), Intensivstation (ICU), Beatmung (MV) sowie den Anschluss an eine externe Sauerstoffanreicherung (ECMO = Extracorporal membran oxygenisation). Weiterhin gibt es noch die Zustände Entlassung (Discharge) und Tod (Death). Abb. 6 zeigt das Ergebnis ihrer Multi-State-Analyse.

Diese Grafik gibt aber keine Information darüber, wie Corona-infizierte Personen das Gesundheitssystem belasten und wie hoch ihre Krankheitsrisiken sind. Diese Graphik sagt nur etwas über COVID‑19 Patienten aus, die in ein Krankenhaus eingewiesen wurden. Infizierte mit asymptomatischen Verläufen merken noch nicht einmal, dass sie infiziert sind.

In der biometrischen Ereigniszeit-Analyse spielen die von Rieg et al. (2020) verwendeten Mehrstadien-Modelle eine herausragende Rolle, vgl. Andersen und Keiding (2002), Beyersmann et al. (2012) sowie Schuhmacher et al. (2013). Gemessen wird hier die Zeit bis zum Eintritt eines neuen Krankheitszustands. Es ist naheliegend, auch für Daten aus einer Längsschnitterhebung solche Multi-State Ansätze zu verwenden, vorausgesetzt, die Zeiten des Übergangs von einem Zustand in einen anderen sind individuell erfasst und dokumentiert.

Verwendet man Daten von Krankenhauspatienten, beruhen alle ermittelten Charakteristika des Verlaufs auf der Schätzung bedingter Wahrscheinlichkeiten. Das bedingende Ereignis in der Studie von Rieg et al. (2020) ist die Klinikeinweisung. Was fehlt, ist eine Übertragung der bedingten Ergebnisse auf die Gesamtbevölkerung. Dieser Übertrag kann vollzogen werden, wenn es gelingt, aus einer Längsschnittstudie, zum Beispiel mit Hilfe der Teilnehmer des SOEP-Panels, die Wahrscheinlichkeit des bedingenden Ereignisses zu schätzen. Ohne auf die möglicherweise etwas komplexere Datenstruktur einzugehen, lässt sich das Vorgehen in einem Mehrstadien-Modell für die Panelstudie veranschaulichen. Wir betrachten zur Erläuterung nur die folgenden Zustände:

$$\boldsymbol{O}-\text{Ausgangszustand}\rightarrow \boldsymbol{I}-\text{Infektion}\rightarrow \boldsymbol{K}-\text{Krankenhaus}$$

Für den Übergang eines Zustands in den darauf folgenden Zustand werden die zugehörigen Ereigniszeiten für die Schätzung benötigt. Dabei wird für den Übergang ***O*** → ***I ***die Kalenderzeit ***t***, für die weiteren ab Infektionsbeginn die Dauer der Infektion ***d*** als Zeitskala verwendet. Aus den erfassten Infektionen wird ***P***_***t***_*** (I)***, die Wahrscheinlichkeit für eine Infektion geschätzt. Diese Schätzung sagt dann etwas über die Dynamik der Infektion aus. Für Infizierte wird der Zeitpunkt der Krankenhauseinweisung zur Schätzung der Einweisungswahrscheinlichkeit ***P(K | I)*** verwendet. Eine Schätzung der unbedingten Wahrscheinlichkeit einer Krankenhauseinweisung kann durch Einsetzen in die einfache Formel für bedingte Wahrscheinlichkeiten ***P***_***t***_*** (K)*** = ***P(K | I) P***_***t***_*** (I) ***bestimmt werden. Der Übergang zu den im Krankenhaus ermittelten Krankheitsrisiken ist somit für die Gesamtpopulation möglich. Eine ausführlichere Herleitung der zugrunde liegenden Schätzverfahren ist in der angegebenen Literatur sehr gut dargestellt; es gibt auch entsprechende Software zum praktischen Einsatz.

Der in Rendtel et al. (2020) ursprünglich vorgeschlagene Längsschnitt-Ansatz als Studiendesign, hätte alle Anforderungen für den Einsatz der Multi-State Methodik ermöglicht. Um das Potenzial des schließlich realisierten Querschittsdesigns der RKI-SOEP Covid-19 Studie zu beschreiben, werden überschlägige Berechnungen der erwarteten Anzahlen von Infizierten und Krankenhauseinweisungen durchgeführt. Diese erwarteten Anzahlen von Ereignissen sind deshalb von großer Bedeutung, weil bei Ereigniszeitanalysen die Varianz von Schätzergebnissen nicht durch die Stichprobengröße, sondern durch die Anzahl eingetretener Ereignisse (bzw. Übergänge) bestimmt wird. Gleiches gilt für die Power von Tests. Wir gehen bei der folgenden Fallzahlabschätzung davon aus, dass für alle Infizierten in der Stichprobe der Infektionszeitpunkt und die Zeitspanne bis zur Krankenhauseinweisung prospektiv oder retrospektiv ermittelt und im Studiendatensatz dokumentiert sind.

### Erwartete Anzahl von Infizierten und Krankenhauseinweisungen

Mit den folgenden Annahmen (5–10 % Infektionsrate in der Studienkohorte sowie 10–20 % Anteil Krankenhausaufenthalte bei Infizierten) lässt sich die erwartete Anzahl von erfassten Übergangszeiten (N total) in der Kohorte abschätzen. (Tab. 2).

**Table 2:** 

N Kohorte	N Infektion	N Krankenhaus
15.000	1050	157
30.000	2100	315

Der Multi-State Ansatz erlaubt auch die Analyse des Einflusses möglicher Risikofaktoren auf die Übergangsraten zwischen den Stadien, also z. B. welchen Einfluss das Alter eines Infizierten auf das Risiko einer Krankenhauspflichtigen Erkrankung hat. Dazu wird in der Regel ein Cox-Proportional- Hazard Modell (Cox 1972) verwendet.

Für einen dichotomen Risikofaktor, z. B. eine Einteilung in zwei Altersgruppen, kann man angeben, welche Werte des zugehörigen Hazard Ratios (HR) statistisch nachweisbar sind. Während für die Schätzung des HR die gesamte Stichprobe verwendet wird, ist für die Varianz nur die Anzahl von Ereignisfällen von Bedeutung. Die Berechnungen gehören zum klassischen Repertoire der Ereigniszeitanalyse und sind in diversen Tools zur Stichprobenplanung implementiert, vgl. z. B. Kohn und Senyak (2021).

Zum Nachweis, welchen Effekt das Alter auf das Einweisungsrisiko hat, wird die Cox Regression verwendet. Wir sehen in Tab. 3, dass auch geringe HR-Werte mit relativ kleinen Fallzahlen statistisch nachweisbar sind.

**Table 3:** 

Hazard Ratio HR	Erforderliche Anzahl N Krankenhaus
2,0	51
1,5	150

Diese überschlägigen Berechnungen zeigen, dass zumindest für die kumulativen Infektionszahlen die in Tab. 2 erwarteten Fallzahlen hoch genug sind, um statistisch abgesicherte Aussagen über Infektionsrisiken im Rahmen der Ereigniszeit Analyse machen zu können.

Dieser Abschnitt zeigt, welches Potenzial ein Längsschnittstudiendesign oder durch Nachbefragung ergänzte Studiendaten einer Querschnittstudie für die Bewertung des Pandemiegeschehens hat, wenn die hier adäquaten Ereigniszeit-Analysen zu Einsatz kommen. Weitergehende Überlegungen zu Adjustierung der Schätzergebnisse und Möglichkeiten zur Konstruktion eines Vorhersagemodells würden den Rahmen dieses Überblicks sprengen.

### Räumliche und zeitliche Intensitäten des Infektionsgeschehens

**Figure 7:**
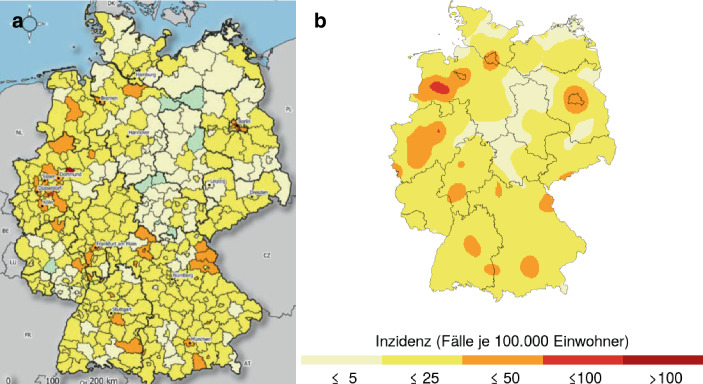

Die Intensität der Corona-Pandemie in Deutschland schwankt rasch mit wechselnden lokalen Hot-Spots, vgl. die Abb. 1 und 2 oben. Hinzu kommt das Problem, dass die Darstellungsweise in den RKI-Tagesberichten die Identifikation von lokalen Clustern eher erschwert denn unterstützt. Hier benutzt der RKI ausschließlich Darstellungen, die jedem Landkreis über seine gesamte Fläche genau einen Inzidenzwert zuordnen. Diese Darstellung über sogenannte Choroplethen erzeugt den visuellen Eindruck, dass das Infektionsgeschehen im Landkreis gleichverteilt ist und an den Grenzen des Landkreises harte Sprünge auftreten, vgl. Abb. 7a die das Infektionsgeschehen am 3. Oktober 2020 am Beginn der zweiten Welle darstellt. Freilich geschieht die Ausbreitung des Corona-Virus in erster Linie über räumliche Nähe, die nicht an Landkreisgrenzen abgeschnitten wird; zum Beispiel am Arbeitsplatz, in der Schule, beim Einkauf, im Restaurant, im öffentlichen Nahverkehr oder im eigenen Haushalt. Dies impliziert die Existenz von lokalen Clustern mit erhöhten Inzidenzzahlen, die nicht gut mit den Kreis- bzw. Landkreisgrenzen beschrieben werden können. Da die getroffenen Eindämmungsmaßnahmen der Corona-Pandemie darauf abzielten, Ansteckungen auch durch Einschränkungen der regionalen Beweglichkeit (Sperrung von Landkreisen, Urlaubzielen, Schließung von Hotels) zu unterbinden, ist auch mit einer gewissen zeitlichen Stabilität dieser Cluster (Hotspots) zu rechnen. Hierbei kann man mehrere Cluster identifizieren, die sich über mehrere Wochen deutlich von ihrer Umgebung durch höhere Inzidenzzahlen abheben. Jedoch bleiben diese Cluster auch über eine zeitliche Aneinanderreihung von zeitlich aufeinanderfolgender Choroplethen verborgen, da diese Cluster in der Regel nicht mit den Landkreisen identisch sind. Der Leser sei hier auf eine Animation im Berliner Tagesspiegel^23^ hingewiesen, die lediglich zufällige Änderungen auf dem Flickenteppich der Landkreise demonstriert. Regionale Trends sind dieser Animation kaum zu entnehmen.

Mit einer anderen Darstellungstechnik lassen sich mit denselben Landkreis-Inzidenzzahlen wesentlich realistischere Ergebnisse erzielen. Hierbei werden die Aggregatszahlen angrenzender Landkreise gemeinsam in die Berechnung von örtlichen Fallzahlen einbezogen. Das Hauptelement ist die Schätzung einer von Kreisgrenzen unabhängigen Dichte von Corona-Infektionen über einen Kerndichteschätzer. Hierbei werden die unbekannten Geokoordinaten von Infektionsfällen aus einer aktuellen Dichteschätzung simuliert. Die Simulation erfolgt so, dass in jedem Kreis nur so viele Fälle simuliert werden, wie das RKI berichtet hat. Insofern sind die Ergebnisse voll mit den RKI-Angaben kompatibel. Abb. 7b zeigt eine alternative Darstellung des Infektionsgeschehens vom 3. Oktober. Deutlich zu erkennen ist ein Infektionscluster um die Stadt Cloppenburg, südwestlich von Bremen. Dieses Cluster kann mehr als 6 Monate über den gesamten Verlauf der zweiten Infektionswelle verfolgt werden. Für die Darstellung der zeitlichen Entwicklung dieser Cluster steht eine spezielle Web-Applikation^24^ zur Verfügung, die täglich aktualisiert wird. Der methodisch interessierte Leser findet eine Darstellung des statistischen Hintergrunds und des benutzten Algorithmus in Groß et al. (2020). Eine Beschreibung der Web-Applikation und ihrer Benutzung findet man in Rendtel et al. (2021). Das RKI gibt seinerseits keine Hinweise auf alternative Nutzungen seiner Datenbasis.

Eine geeignete Darstellung der räumlich-zeitlichen Entwicklung der durch die Gesundheitsämter bestätigten Infektionszahlen gibt auch Hinweise darauf, wo die Anzahl der nicht bestätigten Corona-Infektionen hoch ist. Allerdings kann man hierfür nicht mehr auf RKI-Zahlen zurückgreifen, sondern muss regionale Stichproben ziehen. Die Schätzung der Corona-Dunkelziffern für Landkreise auf der Basis einer Stichprobe ist eine methodische Herausforderung, da hier die Fallzahlen in der Regel zu klein sind, um ein Ereignis, das nach 14 Monaten der Pandemie mit einer Häufigkeit von nur ca. 4,4 % aller Fälle in der Population (3,6 Mio. Corona-Infektionen (kumulativ bis April 2021) auf 82 Mio. Einwohner) auftritt, mit einfachen Methoden zuverlässig zu schätzen. Alternativ kann man natürlich mit weitaus höheren Stichprobenumfängen arbeiten.

Aus diesem Grund starteten nationale repräsentative Corona-Studien mit hohen Fallzahlen, z. B. in Italien mit 150 Tsd. Teilnehmern in 2000 Städten^25^. In der Frühzeit der Corona-Pandemie schienen die noch geringen nationalen Testkapazitäten derartig hohe Stichprobenumfänge zu verbieten. Aber hier wurden schnell große Kapazitätsfortschritte erzielt. So verfügen die mit dem RKI kooperierenden Testlabore mittlerweile über eine wöchentliche Testkapazität von 1,5 Mio. Testungen^26^. Auch werden mittlerweile deutlich preiswertere Tests angeboten.

Für die Corona-Forschung in Deutschland stand aber ein groß angelegter Stichprobenrahmen mit einer entsprechenden Finanzierung nicht zur Verfügung. Außerdem sind die traditionellen akademischen Förderungsquellen wie zum Beispiel die DFG mit ihren Antrags- und Begutachtungsverfahren viel zu langsam, um in kurzer Zeit eine große Corona Umfrage zu ermöglichen.

Aufgrund der gezeigten großen regionalen Unterschiede bei den Inzidenzzahlen erscheint es wünschenswert, auch die Schätzung der Dunkelziffer regional zu differenzieren. Dies ist wegen der geringen Fallzahlen in einem Corona-Survey methodisch aufwändig und nur unter Einsatz von Bayes-Modellen und Vorinformationen möglich. Wir skizzieren hier einen Bayes-Ansatz, in den sowohl die Fallzahlen der durchgeführten lokalen PCR-Tests als auch die Anzahl der nicht getesteten Personen im Kreisgebiet eingehen. Die Nicht-Getesteten erhalten dann den Posteriori Erwartungswert der Positiv Rate zugewiesen. In die Berechnung der Posteriori-Verteilung fließt sowohl eine korrigierte Verteilung der lokalen Positiv-Rate bei den PCR-Tests durch die Gesundheitsämter (GA) ein als auch die Stichprobeninformation. Für die Stichprobeninformation muss gegebenenfalls auf eine höhere Aggregationsebene oberhalb der Kreisebene zurückgegriffen werden.

Zur Schätzung einer lokalen Dunkelziffer unter Ausnutzung aller verfügbaren Informationen kann man einen retrospektiven Fragebogen verwenden, der auch in früheren Seroprävalenzstudien benutzt wird. Dort wird nach früheren Testungen durch das Gesundheitsamt (GA) gefragt. Die möglichen Antworten hierauf sind: Positiver PCR-Test, Negativer PCR-Test und Kein Test durchgeführt. Zusammen mit den Ausgängen des Tests in der Umfrage liefert dies die 6 Felder von Tab. 4.

**Table 4:** 

	Umfrage-Test	
GA = Positiv	Positiv	Negativ
GA = Negativ	Positiv	Negativ
GA = Kein Test	Positiv	Negativ

Wenn nun die Anzahl der Corona-positiven Personen in einem Landkreis (N_(Positiv)) geschätzt werden soll, so ergibt sich diese aus den bereits über das Gesundheitsamt ermittelten Personen N_(GA = Positiv) sowie aus dem positiven Anteil pi_(positiv) der noch nicht über das Gesundheitsamt getesteten Personen multipliziert mit deren bekannter Anzahl N_(GA = Kein Test):

$$\mathrm{N}\_ (\text{positiv})=\mathrm{N}\_ (\mathrm{GA}=\text{Positiv})+\mathrm{N}\_ (\mathrm{GA}=\text{Kein Test})*\mathrm{pi}\_ (\text{positiv})$$

Eine flexible Möglichkeit besteht in einem Bayesianschen Ansatz, der eine lokale Priorverteilung pi verwendet. Beispielsweise kann man eine Beta-Verteilung als a‑Priori Verteilung benutzen, deren Parameter durch die lokale Anzahl der durch das Gesundheitsamt positiv oder negativ Getesteten bestimmt werden. Dies läuft darauf hinaus, den lokalen Positiv-Anteil unter den PCR-Tests, der während der zweiten Welle zwischen 6 und 15 % schwankt, als Vorinformation zu benutzen. Allerdings kann man dagegen einwenden, dass die Positiv-Rate unter Kontaktpersonen und Personen mit Corona-ähnlichen Symptomen über der Positiv-Rate bei einer Testung der gesamten Population liegt, d. h. es ist sinnvoll einen Abschlagsfaktor C zu wählen:

$$\mathrm{E}(\mathrm{pi})=\mathrm{C}*(\text{lokaler Positiv-Anteil PCR-Test})$$

Damit dieser Abschlagsfaktor C aus der Stichprobe geschätzt werden kann, muss er als konstant über alle Landkreise angenommen werden. Somit ergibt sich für die Gesamtpopulation die Schätzung:

$$\mathrm{C}=\text{Positiv-Anteil in Gesamtstichprobe}/\text{Positiv-Anteil in PCR-Tests bundesweit}$$

Es können aber gegebenenfalls noch weitere Informationen in die a‑Priori Verteilung integriert werden. Beispielsweise liefern Konzentrationen von Corona-Viren im Abwasser Hinweise auf die Intensität von lokalen Corona-Infektionen^27^.

Die a‑priori Verteilung wird nun durch die Beobachtungen aus der Stichprobe zu einer Posteriori Verteilung transformiert. Nimmt man für die Beobachtungen aus der Stichprobe eine Binomialverteilung mit einer Wahrscheinlichkeit p_(positiv) für einen positiven Befund aus dem Stichprobentest, so ist die Posteriori Verteilung auf p_(positiv) wieder durch eine Beta-Verteilung gegeben, deren Parameter sich aus der Summe der Parameter der a‑Priori-Verteilung und der Binomialverteilung ergeben. Ist die lokale Fallzahl in der Stichprobe klein, so ist auch deren Einfluss auf die Parameter der Posteriori Verteilung klein. In diesen Fällen wird der Posteriori Erwartungswert von pi_(positiv) ungefähr mit der korrigierten lokalen Positivrate C * N_(GA = positiv) / (N_(GA = positiv) + N_(GA = negativ)) übereinstimmen. Allerdings kann man für die Stichprobeninformation auch eine größere Basis benutzen, indem man auf größere Flächen, etwa Bundesländer, übergeht. Im Extremfall benutzt man die gesamte Stichprobeninformation. Benutzt man eine nicht-informative Prior-Verteilung mit den Verteilungsparametern alpha = beta = 1, so ist der Posteriori Erwartungswert von pi_(positiv) fast identisch mit dem Positiv-Anteil in der Stichprobe. In jedem Fall aber steigt durch diesen Ansatz die geschätzte Anzahl der Corona-Infizierten um den Betrag N_(GA = Kein Test) * pi_(positiv).

Alternativ bieten sich für die Schätzung der regionalen Infektionszahlen auch Small Area Schätzverfahren an, vgl. Münnich et al. (2013). Diese basieren auf einem linearen Prädiktionsmodell zur Vorhersage der Kreis-Infektionszahlen. Hierfür werden bekannte Kovariablen auf Kreisebene benötigt. Die RKI-Zahlen über die gemeldeten Infektionsfälle im Kreis stellen hierfür eine wichtige Information dar. Es können aber auch Informationen zur Altersstruktur oder die Anzahl der Plätze in Pflegeheimen genutzt werden.

Die vom RKI bereitgestellten Infektionszahlen differenzieren innerhalb eines Landkreises nach Altersgruppe und Geschlecht. Dies eröffnet die Möglichkeit, dass oben vorgestellte Bayes-Verfahren auch innerhalb einer Alters‑/Geschlechtsgruppe durchzuführen. Wegen der dann schnell sinkenden Fallzahlen innerhalb von Gruppen, ist man gezwungen die Beobachtungen zu größeren Clustern zusammenzufassen.

Aufgrund einer Intervention wegen datenschutzrechtlicher Bedenken ist es bei der späteren Realisation der RKI-SOEP Studie nicht gestattet worden, Kinder und Jugendliche zu testen. Daher müssen die Fallzahlen bei den PCR-Tests um diese Gruppe reduziert werden, was zwar prinzipiell möglich ist, aber die Analysen komplexer macht und vor allen Dingen die Ermittlung der Dunkelziffern für Kinder und Jugendliche ausschließt.

Die zeitliche Entwicklung der Infektionsfälle und Ansteckungsrisiken ist von hoher Relevanz, insbesondere im Hinblick auf die Einrichtung bzw. Lockerung von Infektionsschutzmaßnahmen. Der stark wechselnde Verlauf der Infektionszahlen ist nicht nur das Resultat der Wellendynamik einer Pandemie, sondern auch Folge von Festtagen mit Familienfeiern, dem Auftreten von neuen ansteckenden Mutationen des Virus oder dem Einsatz von Impfungen. Hier kann man über eine wiederholte Testung einer Stichprobe ein konstantes „Messinstrument“ schaffen, das Veränderungen der Infektionsintensität zuverlässiger anzeigt als die offiziellen Infektionszahlen, die möglicherweise zusätzlich durch wechselnde Testungsstrategien beeinflusst wurden. Hierzu müsste man allerdings die Probanden der Stichprobe relativ häufig testen, was auf Akzeptanzschwierigkeiten bei den Stichprobenteilnehmern stoßen könnte^28^. Die Münchener Regionalstudie KoCo19 hat im Rahmen dieses Konzepts mittlerweile ihre Probanden zum dritten Mal getestet^29^.

Ein Ausweg wäre eine rollierende Stichprobe, wie sie beispielsweise beim Mikrozensus angewendet wird^30^. In diesem Fall wird man die Stichprobe in einzelne Tranchen aufspalten, die zeitlich versetzt getestet werden. Dies würde auch im Falle von Selbst-Tests die Logistik des Versands von 10-Tausenden von Testmaterialien entzerren.

### Sozio-ökonomische Hintergrundvariablen

Die Rekonstruktion der sozialen Bedingungen des Infektionsgeschehens ist ein zentrales Anliegen der Epidemiologie. Beispielsweise können Angaben zum Beruf, zur Größe des sozialen Umfelds oder zur Nutzung des öffentlichen Nahverkehrs Rückschlüsse auf das Ansteckungsrisiko zulassen. Da sich nach der ersten Infektionswelle eine politische Bewegung der sogenannten Corona-Leugner etabliert hat, die dezidiert Hygienemaßnahmen wie Abstand-Halten und Masken-Tragen ablehnen, vermuten einige Autoren, dass man auch aus Angaben zu politischen Präferenzen, ein Ansteckungsrisiko herleiten kann^31^.

## Erhebungskonzepte für Corona-Studien

Während bislang die grundsätzlich interessierenden Merkmale diskutiert wurden, mit der die Corona-Pandemie beschrieben und analysiert werden kann, werden im folgenden konkrete Umsetzungsmöglichkeiten für einen Corona-Survey diskutiert.

### Separate Stichproben vs. Erweiterung einer Basisstichprobe

Schnell und Smid (2020) diskutieren die Survey-Erfordernisse für eine wissenschaftliche epidemiologische Forschung zu Sars-Cov‑2. Sie fordern hierzu insgesamt vier unterschiedliche Erhebungen: Ein Prävalenz-Sample zur Feststellung der Inzidenz-Werte und der Dunkelziffer, eine Panel-Studie zur Erhebung des Verlaufs einer Corona-Infektion, eine Postmortem Studie zur Erforschung der Letalität von COVID‑19 sowie eine sozialwissenschaftliche Studie zur Erforschung von Einstellungen zur Corona Pandemie und ihren sozialen Folgen. Jede dieser vier Studien soll auf einer separaten Erhebung basieren: Das Prävalenz-Sample auf einer zweistufigen Auswahl von Gemeinden und daran anschließend von Personen aus Einwohnermelderegistern, das Panel auf einer Stichprobe von Personen in Quarantäne, die Postmortem-Studie auf einer Obduktion von Personen, die mit Corona – aber eventuell nicht an Corona gestorben sind – sowie schließlich ein sozialwissenschaftliches Panel auf Basis des SOEP, des PASS^32^ oder von SHARE^33^. Hierbei sind das PASS und SHARE Erhebungen für Spezialpopulationen. PASS adressiert Hartz-IV-Empfänger und SHARE Personen über 50 Jahre.

Dieses Programm bedeutet einerseits ein ziemlich großes Erhebungsprogramm mit einem erheblichen finanziellen Aufwand. Hierbei ist die Ergänzung der sozialwissenschaftlichen Panels um Corona-Fragen noch relativ leicht zu realisieren und wurde beim SOEP schon in der Frühphase der Corona-Epidemie implementiert^34^. Andererseits sind die vier von Schnell und Smid vorgeschlagenen Studien unverbunden. Dies stellt einen erheblichen Nachteil dar, da die Ergebnisse medizinischer Tests nicht mit den sozio-demographischen Hintergrundvariablen bzw. mit längeren Beobachtungsintervallen im Rahmen einer Panel-Erhebung verknüpft werden können. Beobachtungen individueller Infektionsverläufe wären so nur retrospektiv möglich.

Selbst wenn eine Testungsstichprobe aus einer bereits existierenden Stichprobe gezogen wurde, ist eine Verknüpfung der beiden Inhalte nicht immer unbedingt sinnvoll bzw. sie hängt von datenschutzrechtlichen Rahmenbedingungen ab – wie zum Beispiel bei der Corona-Deutschland-Studie (Ifo Institut/Forsa 2020). Für diese Studie wurde eine 11.000-er Testungsstichprobe aus einer 30.000-er Telefonstichprobe des Umfrageinstituts Forsa gezogen^35^. Die Testpersonen wurden zweimal (im August und im November 2020) auf Antigene getestet. Im Abschlussbericht (Ifo Institut/Forsa 2020) werden zwar Antworten auf Fragen zur Einstellung zur Corona-Pandemie dokumentiert, diese wurden aber nicht mit den Ergebnissen der Testung auf Individualebene verknüpft. Eine Verknüpfung der Testung mit dem Befragungsteil war offenbar nicht möglich bzw. wurde als sinnlos erachtet. So heißt es im Abschlussbericht (Ifo Institut/Forsa 2020, S. 51), dass aufgrund der geringen positiven-Befunde bei den Testungen „die beabsichtigte Verknüpfung medizinischer Daten mit sozioökonomischen und sozialpsychologischen Faktoren sowie die Verfolgung des Krankheitsverlaufs nicht vorgenommen werden konnten“. Die genauen datenschutzrechtlichen Bedenken wurden in dem Abschlussbericht nicht dokumentiert.

Ein alternativer Ansatz zu Schnell und Smid (2020) basiert auf einer existierenden Stichprobe, einer Basisstichprobe, die durch medizinische Testungen ergänzt wird. In diesem Fall ist die Verknüpfbarkeit des Testergebnisses mit anderen Befragungsinhalten in jedem Fall gewährleistet. Das Konzept hat allerdings auch gewisse Einschränkungen. So ist die statistische Power der Testungen durch die Größe der Ausgangsstichprobe beschränkt. Weiterhin muss das Thema der Basisunterstichprobe in einem sinnvollen Zusammenhang mit dem medizinischen Test stehen. Andernfalls könnte es zu Akzeptanzproblemen bei der Basisbefragung kommen. Spezielle Befragungen haben den Vorteil, dass das Befragungsprogramm auf den thematischen Schwerpunkt genauer eingehen kann.

Die Basiserhebung sollte eine prospektive Wiederholungsbefragung sein, um auch die dynamischen Aspekte einer Infektion abbilden zu können. Bei diesem Ansatz besteht das Risiko, dass bei Akzeptanzproblemen mit der Testung Personen aus der Basisbefragung eine weitere Kooperation auch in der Basisbefragung verweigern, was für die Weiterführung des Basis-Längsschnitts negative Auswirkungen haben würde.^36^

### Überlegungen zur Auswahl der Basisstichprobe

Prinzipiell gibt es zwei Möglichkeiten zur Auswahl der Basisstichprobe: Man kann eine medizinisch orientierte Studie wählen oder eine eher sozialwissenschaftlich orientierte Erhebung.

Für Deutschland ist die NAKO Gesundheitsstudie eine große und auch langfristig angelegte Studie zum umfassenden Gesundheits-Monitoring, vgl. Ahrens et al. (2020). Diese Studie arbeitet mit 18 Studienzentren in acht Regionen zusammen. Dies führt in natürlicher Weise zu einer Clusterung der Stichprobe^37^. Die Testung erfolgt vor Ort in den Studienzentren durch medizinisches Personal, was eine Hürde für die Teilnahme darstellt und zu Selbst-Selektivität führen kann^38^. Auch die regionalen RKI-Studien nutzen ausschließlich Corona-Testzentren vor Ort bzw. DRK-Blutspendezentren. Ähnliche Selektionseffekte wie beim Gang zum Testzentrum dürfte auch der Vorschlag von Schnell und Smid (2020) hervorrufen, die für die Befragten der Prävalenzstichprobe eine Testung beim Hausarzt vorschlagen.

Etwas näher kam die oben bereits genannte Corona-Deutschland Studie ihren Probanden (Ifo Institut/Forsa 2020). Hier kam geschultes medizinisches Personal in die Befragungshaushalte um eine Blutprobe zu nehmen. Damit ist allerdings eine andere Gefahr verbunden: Aufgrund der hohen Ansteckungsgefahr mit SARS-Cov‑2 Viren könnte bei einigen Probanden die Furcht bestehen, dass das Testpersonal das Corona Virus in den Haushalt einschleppt. Auch diese Befürchtung hat ein hohes Potenzial zur Selbstselektion. Hinzu kommt das Problem, dass das Statistikgeheimnis verletzt wird, wenn das Ergebnis eines Tests als akute Infektion dem Gesundheitsamt gemeldet werden muss. Dadurch kann es ebenfalls zu Selbst-Selektionseffekten kommen.

Wählt man als Basisbefragung eine Sozialwissenschaftliche Studie aus, so steht in der Regel kein medizinisch geschultes Personal für die Testung zur Verfügung. Hier muss man auf das Mittel der Selbsttestung zurückgreifen. Damit entfallen alle Selektionseffekte durch den Gang zu einem Testzentrum oder die Angst vor einer Einschleppung von SARS-CoV‑2 in den Haushalt. Allerdings könnte es eventuell Vorbehalte gegen die Zuverlässigkeit der Testergebnisse geben, weil entweder die Tests als solche nicht immer den korrekten Befund ermitteln oder weil die Probanden die Tests nicht sachgemäß anwenden. Doch hier sind bei der Entwicklung von einfach anzuwendenden Corona-Tests im Jahr 2020 große Fortschritte gemacht worden, so dass eine Selbsttestung im Rahmen des Haushaltskontexts eine realistische Alternative zum Einsatz von medizinischem Personal ist. Hierauf wird weiter unten näher eingegangen.

Aus rein methodischer Sicht wäre eine große sozio-ökonomische Stichprobe mit Pflichtteilnahme ideal für eine Basisstichprobe. Dies würde für den Mikrozensus (MZ) sprechen, der eine Erhebung mit Pflichtteilnahme ist. Ferner liefert er als Rotationspanel auch Längsschnittinformationen aus drei vorhergehenden Befragungen und ist zugleich eine Haushaltsbefragung. Allerdings wäre eine Pflichtteilnahme, die per Gesetz angeordnet werden müsste und im Falle von Tests auf akute Infektion das gesetzlich geschützte Statistik-Geheimnis verletzten würde, nur extrem schwer zu realisieren. Bei einer freiwilligen Teilnahme schmilzt der Vorteil der hohen Mikrozensus-Stichprobenzahl von 800 Tsd. Personen im Querschnitt rasch zusammen (vgl. Amarov und Rendtel 2013). Die sogenannte Dauerstichprobe (DSP) des Statistischen Bundesamts rekrutiert sich aus den regelmäßig aus dem MZ ausscheidenden Stichprobenmitgliedern (200 Tsd.) Personen. Wenn diese nach ihrer Bereitschaft gefragt werden, an weiteren Befragungen der amtlichen Statistik teilzunehmen, so erreicht man nur eine Bereitschaft von 15 %, was 30 Tsd. Personen entspricht. Bei der tatsächlichen Rekrutierung für die ersten Wellen von EU-SILC^39^ lag die Teilnahmerate bei 75 %, was 22 Tsd. Personen entspricht. Damit ist man in der Größenordnung der Teilnehmer sozialwissenschaftlicher Erhebungen.

### Auswahlverfahren und Befragungsmodus

Auswahlverfahren für Surveys werden üblicherweise unter Kostengesichtspunkten als auch Genauigkeitsaspekten diskutiert. Für das Auswahlverfahren von Corona-Erhebungen existiert auch ein zeitlicher Aspekt. Die Dynamik der Corona-Pandemie macht schnelle Reaktionen wünschenswert. Der Aufschwung der zweiten Infektionswelle zu zuvor unerreichten Infektions- und Sterbezahlen fand in nur drei Monaten statt. Virusmutationen, deren Eigenschaften noch unbekannt sind, verbreiten sich mit sehr hoher Geschwindigkeit. Hier ist die Ziehung von neuen Stichproben (aus Survey-methodischer Sicht idealerweise über die Einwohnermeldeämter^40^) und der Aufbau einer neuen Feldorganisation grundsätzlich deutlich langsamer als der Versand von Selbsttests an eine bestehende Stichprobe und die Aufbereitung der Teilnahme- und Testergebnisse durch ein eingeübtes Survey-Team. Unter dem zeitlichen Aspekt wie auch dem Logistik-Aspekt ist die Nutzung einer bestehenden Stichprobe die grundsätzlich günstigere Variante.

Der wahrscheinlich größte Vorteil der Rekrutierung über eine Basisstichprobe besteht in den Möglichkeiten der Ausfallanalyse bzw. der Analyse der Selektivität der an Testungen teilnehmenden Personen. Eine Basisstichprobe enthält typischerweise viele Merkmale, die für eine Analyse der zu erwartenden Teilnahmeausfälle bei der Testung genutzt werden können. Bei einer Auswahl über ein Einwohnermeldeamt, die Schnell und Smid (2020) für ihre Prävalenzerhebung vorschlagen, ist man deutlich reduzierter in der Wahl seiner Merkmale für die Ausfallanalyse. Hier ist man auf Alter, Geschlecht und Familienstand und den Wohnort angewiesen. Bei Telefonstichproben, die für die Auswahl der Corona-Bund-Studie durch FORSA realisiert wurde, gibt es kaum Möglichkeiten zur Kontrolle der hohen und vermutlich selektiven Ausfallraten. Insbesondere gibt es bei den vielen Mobilfunknummern keinerlei Möglichkeit einer regionalen Kontrolle, was bei repräsentativen Aussagen zur Verbreitung von Corona-Infektionen ein wichtiger Aspekt ist. Auch liefern Telefonstichproben ausschließlich Personenstichproben, so dass hier ein Haushaltskontext nicht beobachtet wird. Zwar ist es prinzipiell möglich, über eine Telefon- oder Einwohnermeldeamtsstichprobe auch Haushalte auszuwählen. In diesem Fall ist die Auswahlwahrscheinlichkeit allerdings proportional zu der Anzahl der Haushaltsmitglieder, was eine Designgewichtung erforderlich macht und damit bei gleichen Stichprobengrößen Genauigkeitsverluste der Ergebnisse impliziert.

### Ethische und Datenschutz-Aspekte

Wenn bei Studien virologische und serologische Tests zum Einsatz kommen, gelten die Regeln für medizinische Beobachtungstudien, die u. a. Aufklärung und Einverständnis der Teilnehmenden sowie besondere Sorgfalt beim Datenschutz erforderlich machen. Zusätzlich müssen klar definierte Studienziele und deren Erreichbarkeit, z. B. die statistische Güte geplanter Analysen, unter ethischen Aspekten angemessen belegt werden. Derartig weitreichende Regeln sind bei sozialwissenschaftlichen Befragungen bislang nicht gegeben.

Ein besonderer datenschutzrechtlicher Aspekt ist durch die Mitteilungspflicht eines positiven PCR-Tests an das Gesundheitsamt gegeben^41^. Dadurch wird die völlige Anonymität der Erhebung partiell aufgehoben. Der Weitergabe ihres Namens und ihrer Adresse müssen die Stichprobenmitglieder deswegen explizit zustimmen. Allein die schriftliche Zustimmungs-Notwendigkeit kann zu selektiven Teilnahmeausfällen führen, was die Ausfallanalyse auf Basis möglichst vieler individuellen Angaben umso wichtiger macht. Hinzu kommen Anreizgesichtspunkte. Falsch positive Testungen sind aufgrund der Mitteilung an das Gesundheitsamt pflichtgemäß mit einer Quarantäne verbunden. Dieses Risiko könnte ökonomisch abschreckend wirken. Dass im Falle einer angeordneten Quarantäne die Gesundheitsämter Ersatzzahlungen leisten^42^, ist wenig bekannt. Umgekehrt wird ein falsch negativer Befund die Befragungsteilnehmer in eine irrtümliche Sicherheit vor einer Corona Erkrankung versetzen. Dies kommt jedoch bei den bestehenden Tests sehr selten vor. Letztendlich sind dies unvermeidliche Risiken der Nutzung medizinischer Tests, die auch außerhalb der Befragung jeder Mitbürger im Alltag trägt (vgl. auch z. B. Lampert und Santos-Hövener 2020, Abschn. 4.6).

### Datenzugang und Datennutzung

Die Daten und die Veröffentlichungsrechte an den Daten sind in der Regel im Eigentum des Finanziers einer Studie. Dieser ist bei Corona-Studien häufig das Bundesministerium für Gesundheit. Damit ergeben sich auch Einflussmöglichkeiten auf den Inhalt und die Ergebnisse der Auswertungen. Bei Veröffentlichungen in wissenschaftlichen Zeitschriften wird häufig eine Erklärung zur Unabhängigkeit der Forschungsergebnisse und ihrer Publikation von den Finanziers der Studie erwartet. Darin heißt es: „No rule in study design, data collection and analysis, decision to publish, preparation of manuscript“, vgl zum Beispiel die Erklärung in Wagner et al. (2021) zur Tirschenreuth-Studie.

Eine Koppelung der Testung mit sozialwissenschaftlichen Befragungen, die als Basis für wissenschaftliche Forschungsprojekte dienen soll, steht damit in einem Spannungsverhältnis zu den Einschränkungen einer über ein Ministerium finanzierten Forschung. Diese Sachverhalte müssen im Rahmen von Kooperationsverträgen geregelt werden, was derartige Kooperationsprojekte nicht einfacher macht und den Start von derartigen Projekten verzögern kann.

Hinzu kommt ein spezifischer Aspekt der Corona-Pandemie. Aufgrund der großen regionalen Unterschiede der Corona-Infektionen ist der Zugang zu Informationen unterhalb der Kreisebene wichtig zur Beurteilung des Einflusses lokaler Gegebenheiten auf das Infektionsgeschehen. So veröffentlichte beispielsweise der Berliner Senator für Gesundheit eine Studie^43^ über den Einfluss von soziodemographischen Merkmalen und dem Wohnumfeld auf das Corona-Infektionsrisiko. Zur Verfügung standen aber lediglich die RKI-Zahlen über die Aggregate der 12 Berliner Stadtbezirke. Ein angeforderter Datenzugang auf einer niedrigeren Analyseebene, der in diesem Fall wünschenswert war und von den Autoren der Studie auch angefordert worden war, konnte wegen Überlastung und prinzipieller Datenschutzbedenken nicht realisiert werden. Diese Episode weist auf einen Mangel hin. Es gibt am RKI zwar ein Forschungsdatenzentrum (FDZ), das aber keinen Zugang zu Gesundheitsdaten unter erhöhten Sicherheitsstandards für die interessierte Wissenschaft ermöglicht. Man hat sich mit den im Internet zur Verfügung gestellten Kreisdaten zu begnügen.

Unter diesem Aspekt ist eine Integration einer repräsentativen Corona-Testung in eine Studie mit einem Forschungsdatenzentrum, das wie beispielsweise das SOEP-Forschungsdatenzentrum Analysen mit kleinräumigen Kovariaten, zum Beispiel auf Postleitzahlebene, in einem besonders geschützten Bereich routinemäßig ermöglicht, ein wichtiger Schritt zu einer verbesserten epidemiologischen Forschung.

## Selbsttestung auf eine akute Sars-Cov-2 Infektion und auf Sars-Cov-2 Antikörper

Selbsttestungen auf eine akute Corona-Infektion über einen Nasen- und Rachenabstrich und die Entnahme einiger Bluttropfen aus der Fingerkuppe auf Löschpapier zum Testen auf Corona Antikörper sind seit der zweiten Jahreshälfte 2020 zugelassen. Einzelheiten über die in der RKI-SOEP-Stichprobe verwendeten Tests werden detailliert in dem Studienprotokoll der RKI-SOEP-Studie (Hoebel et al. 2021a) beschrieben.

Für diese medizinischen Tests sind zwei Parameter besonders wichtig.

- Die Sensitivität eines Tests ist seine Fähigkeit medizinisch relevante Merkmale korrekt zu identifizieren. Ein Test mit 100 %-iger Sensitivität würde alle Getesteten mit akuter COVID‑19-Infektion erkennen. Ein Test mit 80 % Sensitivität erkennt 80 % der Infektionen (*richtig-positiv*), aber 20 % Infizierte blieben unentdeckt (*falsch-negativ*).
- Die Spezifität eines Tests bezieht sich auf seine Fähigkeit, die Patienten ohne das relevante Merkmal korrekt zu identifizieren. Ein Test mit 100 %-iger Spezifität klassifiziert alle Probanden ohne COVID‑19-Infektion korrekt als nicht infiziert. Ein Test mit 80 % Spezifität identifiziert 80 % der nicht-infizierten Probanden als Testnegativ (*richtig-negativ*), aber 20 % der Probanden ohne Infektion würden fälschlicherweise als Testpositiv (*falsch-positiv*) klassifiziert.

Für die Berechnung von Prävalenzen müssen beide Fehlerarten berücksichtigt werden. Aus statistischer Sicht ist ein Bayesianischer-Ansatz sinnvoll, der aus fehlerbehafteten Messungen über ein Latent Class Modell valide Schlüsse auf den wahren Krankheitsstatus zieht (vgl. Hartnack et al. 2020).

Wird ein Mund/Nase-Abstrich in den Laboren des RKI oder bei anderen zugelassenen Laboren mit einem PCR-Test analysiert, liefert dieses Testverfahren aufgrund einer zweifachen Testung (auf zwei unterschiedliche Genom-Abschnitte des Virus) eine fast 100 %-ige Spezifität. Allerdings sind die SARS-CoV‑2 Viren nur in einem geringen Zeitfenster über einen Mund/Nase-Abstrich nachweisbar^44^, was die Sensitivität senkt. Die Spezifizität wird mit 95 % angegeben, d. h. etwa 5 % der Beprobten wird fälschlich in Quarantäne geschickt. Die Qualität des Abstrichs durch die getesteten Personen selbst, die in der Regel Laien sind, ist laut Studienprotokoll mit derjenigen durch geschultes Personal vergleichbar.

Ein Test auf Antikörper kann mit Hilfe getrockneter Blutstropfen auf Löschpapier erfolgen. Ein Vorteil der getrockneten Blutstropfen ist vor allem der minimal invasive Eingriff zur Gewinnung der Probe, für den kein Fachpersonal benötigt wird. Die sehr geringe Blutmenge (es genügt bereits ein Tropfen von ca. 30 µL, für eventuelle Nachtestungen sollten jedoch nach Möglichkeit mehrere Tropfen gewonnen werden) ermöglicht auch – im Grundsatz – die einfache Beprobung von Kindern. Außerdem sind die Antikörper gegen SARS-CoV‑2 in dem getrockneten Blut über mehrere Wochen stabil. Die Probe kann so unkompliziert bei Raumtemperatur transportiert werden und stellt ein sehr geringes Infektionsrisiko dar, was den normalen Postversand als freigestellte medizinische Probe ermöglicht.

Ein Test auf Antikörper kann zum Beispiel in einem zentralen Labor eines kommerziellen Anbieters durchgeführt werden. Für den in der RKI-SOEP-Studie verwendeten kommerziellen Labortest „Anti-SARS-CoV‑2 ELISA (IgG)“ von Euroimmun wird eine Sensitivität von 99,4 % und eine Spezifizität von 88,3 % angegeben; d. h. es werden etliche Probanden falsch positiv getestet. Ein Quarantäne- bzw. Ethik-Problem ergibt sich dadurch nicht, da es ja nicht um eine akute Infektion geht. Aber die unter 100 % liegende Spezifizität und Sensitivität müssen bei der Schätzung der kumulativen Dunkelziffer berücksichtigt werden.

## Das Sozio-oekonomische Panel (SOEP) als Basis für eine Corona-Studie

Im vorliegenden Abschnitt wird geschildert, wie das oben beschriebene Konzept der Nutzung einer bestehenden Basisstichprobe vom RKI mit Hilfe der Längschnittstudie Sozio-oekonomische Panel (SOEP) umgesetzt wird. Das Sozio-oekonomische Panel (SOEP) ist in der Sprache der Epidemiologie eine multidisziplinäre Multi-Kohortenstudie, die im jährlichen Rhythmus seit 1984 in Deutschland durchgeführt und zu diesem Zweck im Rahmen der Leibniz-Gesellschaft am Deutschen Institut für Wirtschaftsforschung (DIW Berlin) von Bund und Ländern gefördert wird, vgl. Goebel et al. (2019).

### Fallzahlen und Erhebungseigenschaften

Im Jahr 2020 wurden in verschiedenen Teilstichproben des SOEP (im SOEP-Core und dem SOEP-Innovation Sample), die insgesamt bevölkerungsrepräsentativ sind, für über 40.000 Personen Daten erhoben. Eine Altersbeschränkung existiert nicht, während z. B. in der NAKO-Gesundheitsstudie nur Volljährige einbezogen sind, die beim ersten Untersuchungszeitpunkt nicht älter als 69 Jahre waren. 2020 umfasste das SOEP ca. 11 Tsd. Personen in Ostdeutschland und 38 Tsd. in Westdeutschland. Davon waren 10 Tsd. unter 18 Jahren, 18,5 Tsd. zwischen 18 und 49 Jahren und 20,5 Tsd. Personen älter als 50. Zentrales Merkmal des SOEP ist, dass über alle Haushaltsmitglieder Informationen im Längsschnitt vorliegen. Alle erwachsenen Haushaltsmitglieder sowie im Haushalt lebenden Kinder ab dem 11. Lebensjahr werden direkt befragt. Informationen über im Haushalt lebende Kinder unter 11 Jahren werden über die Eltern ermittelt^45^.

Ein Problem stellen die Personen in Alters- und Pflegeheimen dar. Dieser Bereich ist gerade für die Bestimmung der Corona-Inzidenzen von besonderer Bedeutung, da hier hohe Infektionszahlen beobachtet werden^46^. Zwar werden im Rahmen des längsschnittlichen Weiterverfolgungskonzepts des SOEP auch Personen mit Umzügen in Alters- und Pflegeheime erfasst, doch insgesamt ist die Fallzahl zu gering um Rückschlüsse auf diese Bevölkerungsgruppe machen zu können.

Zum Zeitpunkt der ersten Corona Welle wurde von April bis Juni 2020 bei rund 7000 SOEP-Haushalten eine telefonische Befragung zur Corona-Krise durchgeführt (SOEP-CoV) (vgl. Kühne et al. 2020). Die Befragung beschäftigte sich mit dem Thema, wie die Corona-Krise (insbesondere die landesweit verfügten Beschränkungen) den Alltag der Menschen in Deutschland geprägt hat und wie diese mit der Situation umgegangen sind. Außerdem wird mit den SOEP-CoV Daten untersucht, welche mittel- und längerfristigen wirtschaftlichen und sozialen Folgen durch die Pandemie zu erwarten sind. Dabei geht es zum Beispiel um die Auswirkungen auf die gesundheitlichen und ökonomischen Ungleichheiten zwischen verschiedenen Bevölkerungsgruppen, um die psycho-sozialen Folgen für Einzelne sowie um den gesamtgesellschaftlichen Zusammenhalt. Die Befragung lieferte zudem Informationen über die grundsätzliche Teilnahmebereitschaft an Infektions- und Immunitäts-Tests. Insgesamt haben ca. 90 % aller Befragten ihre grundsätzliche Bereitschaft für eine Teilnahme an einer Testung (Rachenabstrich und Antikörpertest) angezeigt. Ebenso zeigten sich 90 % der am SOEP-CoV teilnehmenden Eltern bereit, ihre Kinder im Rahmen einer Selbstbeprobung zu testen.

### Potenziale einer Coronavirus-Testung im Rahmen des SOEP

Der bereits vorliegende Datenbestand des SOEP bietet in Verbindung mit einer Testung der Mitglieder der SOEP Stichprobe auf eine akute Covid-19-Infektion sowie auf Antikörper im Hinblick auf eine überstandene Infektion über eine eventuelle aktuelle Berichterstattung hinaus vor allem wissenschaftlich relevante analytische Möglichkeiten. Denn hier sind bereits Informationen über Personen und ihre Haushalte verfügbar, die in den vorangegangenen Erhebungswellen zur sozio-ökonomischen Situation, den individuellen oder haushaltsbezogenen Lebensumständen sowie den subjektiven Befindlichkeiten (inklusive selbstberichteten Gesundheitszuständen) für den jeweiligen Erhebungszeitpunkt erfragt wurden (Schupp 2012; Giesselmann et al. 2019) und es werden künftig weitere Informationen, so auch zu den Folgen der Pandemie, erhoben. Für die Mitglieder dieser Studie kann auf eine retrospektive Erhebung von Informationen zur Analyse des Covid-19-Geschehens verzichtet und die damit verbundenen methodischen Probleme und Verzerrungen vermieden werden. Der Einfluss von Lebensumständen und des Gesundheitsstatus auf das Auftreten von Infektionen sowie deren Dynamik kann mittels statistischer Modellierung über den Lebenslauf ermittelt werden. Die sich seit dem Sommer 2020 entwickelnde Bewegung der Corona-Leugner kann ebenfalls gut über frühere Antworten zu Sorgen, zur Parteipräferenz und zur politischen Partizipation analysiert werden (vgl. Rohrer et al. 2017; Meyer et al. 2019).

Mit einem laufenden prospektiven Panel ergibt sich automatisch die Möglichkeit, Folgeeffekte bestimmter Ereignisse in der Zukunft zu identifizieren. Da die Haushalte und ihre Personen auch in weiteren SOEP-Befragungswellen über die nächsten Jahre hinweg regelmäßig befragt werden, können auch langfristige Effekte einer Corona-Infektion, wie etwa Immunität durch Antikörper und deren zeitliche Entwicklung oder Krankheitsverläufe und -folgen, beobachtet und analysiert werden. Für SOEP-Befragte, die eine Infektion – ob symptomatisch oder asymptomatisch – durchgemacht haben, können gezielt Fragen gestellt werden (zum Ansatz des „dependent interviewing“ vgl. Kühne et al. 2020, S. 198).

Darüber hinaus eröffnet das Design einer Haushaltsbefragung, wie das des SOEP, die Möglichkeit, Infektionen bei Familienmitgliedern und Virusübertragungen innerhalb des Haushalts genauer zu rekonstruieren (vgl. Döhla et al. 2020) – wodurch man beispielsweise die Frage nach der Rolle von Kindern in Infektionsketten beantworten kann^47^.

Insgesamt bietet das SOEP somit die Möglichkeit einer prospektiven Studie^48^, und zwar nahezu exakt wie dies die WHO empfiehlt: d. h. im Haushaltskontext einschließlich der Kinder und Hochaltriger und als Längsschnittstudie so früh wie möglich im Verlauf der Pandemie^49^. Zwar erforderte der Planungszeitraum einen Starttermin nach dem September 2020, also nicht ganz am Anfang der Pandemie. Doch dieser Termin fiel genau mit dem Beginn der zweiten Corona-Welle zusammen^50^.

### Durchführung der Tests im SOEP

Im Kontext der üblichen Abläufe der SOEP-Erhebungen sind die Abwicklung der Tests auf Antikörper und die Rachenabstriche über einen professionellen medizinischen Dienst nicht zielführend. Dafür sind zwei Argumente entscheidend: Ein „logistisches“ und ein „Survey-methodologisches“.

Der Einsatz eines medizinischen Dienstes oder medizinisch ausgebildeten Personals ist aufgrund der für die Repräsentativität des SOEP zentralen breiten regionalen Streuung der Stichprobe nur sehr schwer umsetzbar. So wäre die Koordinierung der Testtermine zwischen medizinischem Dienst/Personal und Befragten – letztlich auch aufgrund der räumlichen Distanzen – sehr aufwändig und kaum umsetzbar – nicht zuletzt auch aufgrund der dadurch entstehenden Kosten. Die Koordinierung gesonderter Termine in externen Einrichtungen oder Besuche im Haus bedeuten einen nicht zu vernachlässigenden Aufwand auch auf der Seite der Befragten, wobei Besuche im Haus auch teilweise im Widerspruch zu den Corona-Kontaktbeschränkungen stünden. Insgesamt wäre mit vielen und vor allem sozial selektiven Ausfällen zu rechnen.

Das Survey-methodologische Argument baut auf der Überlegung auf, dass die Teilnahme an einer Testung für die SOEP-Befragten möglichst niederschwellig sein muss, damit eine möglichst hohe Testbereitschaft erreicht werden kann. Eine Testung in medizinischen Zentren, wie dies beispielsweise im Rahmen der NAKO-Gesundheitsstudie vorgenommen wird, wäre für die Haushalte mit Kosten in Form von Zeit verbunden, die sehr wahrscheinlich zu einer systematischen Verzerrung der Stichprobe führen würden (selbst wenn die Wegekosten erstattet würden), da der zu erbringende Aufwand für unterschiedliche Haushaltstypen unterschiedlich hoch ist (regionale Nähe, Anzahl an Personen, Aufwand der Terminvereinbarung aufgrund eigener Erwerbstätigkeit etc.). Zudem ist davon auszugehen, dass die verschiedenen Haushalte auch unterschiedlich bereit sind, diesen Aufwand in Kauf zu nehmen (z. B. ist für ältere und weniger mobile Befragtengruppen mit einem höheren individuellen Aufwand zu rechnen als bei mobilen Gruppen). Vor diesem Hintergrund sollte eine Corona-Beprobung möglichst niederschwellig, d. h. von Befragten selbst durchgeführt werden können. Nur dann ist eine erhöhte Teilnahmequote und eine mit Blick auf relevante Merkmale (wie Erwerbsstatus, Alter, Größe des Haushalts, Erreichbarkeit medizinischer Einrichtungen etc.) geringe Stichprobenselektivität zu erreichen.

Der Vorschlag von Schnell und Smid (2020), die Befragten sollten eine Testung bei ihrem Hausarzt vornehmen, ist keine sinnvolle Alternative. Denn auch hier entsteht auf Seiten der Befragten ein Koordinierungs- und Zeitaufwand, um Termine abzustimmen und diese dann auch wahrzunehmen. Hinzu kommt, dass eine Testung – zur Ausschöpfung des analytischen Potentials des SOEP – für alle Haushaltsmitglieder vorgenommen werden sollte, was gerade für Mehrpersonenhaushalte den Aufwand weiter erhöhen würde. Vor diesem Hintergrund – Durchführbarkeit und Kosten einerseits, Effekte auf die Teilnahmebereitschaft durch hohen Koordinierungs- und Zeitaufwand auf Seiten der Befragten andererseits – ist allein die „Beprobung“, wie das Testen im Medizinerjargon genannt wird, durch die Befragten selbst die realistische und zielführende Methode.

Nach einer ersten Basis-Beprobung können mit der SOEP-Stichprobe – ggf. in regelmäßigen Abständen – weitere Beprobungen auf eine akute bzw. überstandene Corona-Infektion vorgenommen werden. Dabei muss eine derartige Wiederholung der Testung gut motiviert werden, da sonst Verluste durch Panel-Attrition eintreten können^51^.

### Erfahrungen des SOEP mit Selbstbeprobungen

Das SOEP erhebt im Rahmen einer Selbstauskunft der Befragten seit 1984 gesundheitlich und medizinisch relevante Daten im Haushaltskontext (Schupp 2012); seit 2006 wird auch alle zwei Jahre die „Handgreifkraft“ als Indikator für körperliche Fitness physisch gemessen, vgl. Hank et al. (2008), Fuchs und Scheidt-Nave (2016).

Das SOEP verfügt auch bereits für das Einsammeln von Körpermaterial einschlägige Erfahrungen. In einem Pretest-Panel wurden im Jahr 2008 für einige hundert Fälle von Erwachsenen Speichelproben für die Analyse bestimmter Loci im Genom mit Hilfe einer Interviewer-gestützten Selbstbeprobung gewonnen (vgl. Schonlau et al. 2010). In der SOEP Innovations-Stichprobe wurden zudem 2015 (vgl. Gerstorf und Schupp 2016) sowie 2019 und 2020 etwa 2750 Speichelproben ebenfalls mit Hilfe einer Interviewer-gestützten Selbstbeprobung (Swabs) gewonnen. Dabei wurden 2019/20 nicht nur Proben für Erwachsene gewonnen, sondern auch von Kindern, denen die Eltern die Probe entnahmen, um das Genom von Familien (Trios) analysieren zu können. Es liegen im SOEP somit hinreichende Erfahrungen bei der Sammlung von Speichelproben vor, einschließlich der Berücksichtigung der Forschungsethik und des nicht unkomplizierten Datenschutzes^52^.

Unabhängig von den oben bereits genannten Argumenten für eine Selbstbeprobung zeigen die bisherigen Erfahrungen des SOEP somit auch, dass die Einschaltung eines medizinischen Dienstes bzw. medizinischen Personals kein Muss bei der Erhebung von Bioproben in der SOEP-Stichprobe ist (vgl. auch Kühne et al. 2020, S. 197). Das SOEP hat allerdings – ebenso wie alle anderen sozialwissenschaftlichen oder amtlichen Erhebungen – keine Erfahrungen mit dem unumgänglichen Bruch des Statistikgeheimnisses, wenn ein PCR-Test auf akute Infektion von einem zugelassenen Labor durchgeführt wird. Zugelassene Labore müssen laut Infektionsschutzgesetz akute Infektionen an das Gesundheitsamt personenbezogen melden (vgl. Abschn. 3.4 oben).

### Teilnahmemotivation

Die Beteiligung an einer im Rahmen des SOEP durchgeführten Studie ist grundsätzlich freiwillig, wobei die „informierte Zustimmung“ eine Grundvoraussetzung für die Teilnahme an der Studie ist. Zudem müssen alle Aspekte des Schutzes individueller Daten berücksichtigt werden. Der entscheidende Unterschied zu neu aufgesetzten Stichproben (im Querschnitt oder Längsschnitt) besteht im Fall einer SOEP-Studie darin, dass die Haushalte bzw. Personen bereits über mehrere Erhebungswellen Erfahrungen mit der Studie haben. Damit besteht ein gewisses Vertrauensverhältnis und auch eine gewisse Identifikation mit dem SOEP, was wiederum die Bereitschaft zur Teilnahme an einer Beprobungsstudie erwartungsgemäß befördert. Zumindest kann man eine deutlich geringere Teilnahmeschwelle voraussetzen als im Fall einer Erhebung mit Erstkontakt zwischen der Forschungseinrichtung, dem Feldinstitut und den Befragten.

Eine SOEP-basierte Corona-Virus-Beprobungsstudie kann also auf einen Vertrauensvorschuss auf Seiten der Befragten aufbauen, der in anderen Fällen unter normalen Umständen nicht besteht und erst aufgebaut werden müsste. Dies gilt insbesondere auch deshalb, weil die Einhaltung datenschutzrechtlicher Regelungen im SOEP den Befragten hinreichend kommuniziert wird, die Befragten mit der Einhaltung dieser Regelungen durch das SOEP auch hinreichende Erfahrungen haben und dementsprechend die Grundlage für die künftige Kooperationsbereitschaft geschaffen wurde. Wie oben ausgeführt stellt die unumgängliche Meldung einer akuten Infektion an das zuständige Gesundheitsamt allerdings eine Herausforderung dar.

Im Sommer 2020 waren kommerzielle Corona-Selbsttests noch nicht auf dem Markt. In dieser Situation war die Möglichkeit, dass die SOEP-Teilnehmenden ihren Antikörperstatus und damit vermutete Immunität gegen das SARS-CoV‑2-Virus erfahren konnten, ein Motivationsanreiz zur Teilnahme an der Beprobung. Allerdings hat sich dieser Vorteil seit dem Frühjahr 2021 mit kostenlos angebotenen Massentestungen beziehungsweise preiswert in Supermärkten erhältlichen Selbsttests aufgelöst. Weiterhin hat sich seit dem Sommer 2020 eine Bewegung von Corona-Leugnern gebildet, die aus unterschiedlichen Motiven die Maßnahmen zur Eindämmung der Corona-Epidemie ablehnen, beziehungsweise eine Corona-Infektion für eine normale Grippe-ähnliche Erkrankung halten. Da diese Teilpopulation auch im SOEP vertreten ist, ist damit zu rechnen, dass es Schwierigkeiten gibt, diesen Personenkreis zur Teilnahme an der Beprobung zu gewinnen. Im Gegenteil, es könnte sogar zu einem Abbruch der Kooperation für die folgenden SOEP-Befragungen kommen.

### Nonresponse-Gewichtung

Ein großer Vorteil der Nutzung des SOEP als Stichprobenbasis für eine freiwillige Studie zur Testung auf Covid-19 und Antikörper gegen Covid-19 ist die Möglichkeit, den zu erwartenden nicht zufälligen Ausfall in der Stichprobe statistisch durch eine geeignete Gewichtung bzw. Nonresponse-Modellierung über die Merkmale aus dem SOEP quantifizieren und somit kontrollieren zu können. Im Gegensatz zu Stichproben aus dem Einwohnermeldeamt, die lediglich Alter, Geschlecht und den Familienstand als Kontrollmerkmale benutzen können, ergibt sich hier die Möglichkeit, sehr spezifische Merkmale zu nutzen, wie zum Beispiel den Gesundheitsstatus der Person oder die Anzahl der Krankenhausbesuche. Auch die Größe des sozialen Umfelds kann als Kontroll- bzw. Gewichtungsvariable genutzt werden.

### Datennutzung

Ein großer wissenschaftlicher Vorteil der Nutzung der SOEP-Stichprobe zur Testung auf akute Infektion und Antikörper ist die Verknüpfungsmöglichkeit mit den bereits im Längsschnitt vorliegenden Informationen über die Personen und ihre Haushalte. Die Testergebnisse werden dementsprechend dem existierenden Datenbestand des SOEP hinzugefügt und damit Teil des regulären Datenangebots des SOEP für wissenschaftliche Zwecke. Dies bedeutet auch, dass sie für die Scientific Community nutzbar sind. Bei der Aufbereitung, Dokumentation und Bereitstellung dieser Daten kann auf die bestehenden Infrastrukturen und internen Datenproduktionsprozesse des SOEP zurückgegriffen werden.

Da gerade durch die Verknüpfung von Sozial- und Gesundheitsdaten ein hohes datenschutzrechtliches Risikopotential besteht, muss sichergestellt werden, dass auch in der Nachnutzung der Daten keine Identifikation der Befragten vorgenommen wird. Das SOEP hat für derartige datenschutzrechtlich hoch sensible Forschungsdaten besondere Nutzungsmodelle entwickelt, wobei hoch sensible Informationen nur vor Ort im Forschungsdatenzentrum des SOEP (FDZ SOEP) unter besonderen Sicherheitsvorkehrungen genutzt werden können. Genau dieses Modell ist in dem vorliegenden Fall anzuwenden.

## Die Realisierung der SOEP-RKI Stichprobe

Zur Erhebung von Daten zum Vorliegen von Antikörpern (IgG) gegen das Corona-Virus hat das Robert-Koch-Institut eine Kooperation mit dem SOEP vertraglich vereinbart. Die „RKI-SOEP Studie“ ging am 2. Oktober 2020 mit einer Bruttostichprobe von 31.677 erwachsenen Personen in 19.573 Haushalten, die jährlich im Rahmen des SOEP (Core und IS) befragt werden, ins Feld. Nicht eingeschlossen in diese Studie wurden die Haushalte der IAB-BAMF-SOEP Geflüchteten-Befragung, da nicht zuletzt Sprachprobleme eine niedrige Teilnahmebereitschaft und besondere Ethikprobleme hätten erwarten lassen.

Allen Personen in der Bruttostichprobe wurden Päckchen mit Materialien zur Selbstbeprobung auf IgG Antikörper (Trockenbluttests) und auf eine derzeitige Infektion mit dem SARS-CoV‑2 Virus (PCR Tests mit Mund-Nase-Abstrich) per Post zugeschickt. Zusätzlich erhielten alle Befragungspersonen Informationsmaterial, ein Datenschutzblatt, eine Einwilligungserklärung und einen 1‑seitigen Kurzfragebogen zu akuten oder bereits durchgestandenen Corona-Infektionen, der Teilnahme an PCR-Tests, zu Symptomen und Corona-bedingten Krankenhausaufenthalten (vgl. Brix et al. 2021, S. 7ff).^53^

Der Versand aller Materialien und Informationsunterlagen erfolgte in vier zeitlich versetzten Tranchen, um die Logistik organisatorisch bewältigbar zu machen. Auch mit mehr finanziellen Mitteln wäre eine einstufige Feldarbeit nur schwer bewältigbar gewesen, da zur Erstellung und Qualitätssicherung der Erhebungsmaterialien, für eine Hotline zur Beantwortung von Fragen der SOEP Panellisten sowie der Übermittlung der Testergebnisse nur eine beschränkte Anzahl an geschulten und erfahrenen Mitarbeitern zur Verfügung stand (vgl. auch Brix et al. 2021, S. 18ff).

Die ersten drei Tranchen stellen eine zufällige Zerlegung der Menge der Haushalte der Bruttostichprobe dar; nicht einbezogen wurden die beiden reinen Migrationsstichproben M1 und M2 des SOEP, die die Basis für Tranche 4 bildeten^54^. Die erste Tranche umfasste 50 % aller in den ersten drei Tranchen eingesetzten Fälle, die zweite und die dritte Tranche jeweils 25 %.

Tranche 1 wurde am 2. Oktober 2020 ins Feld gegeben, Tranche 2 am 27. Oktober 2020 und Tranche 3 am 10. November 2020. Der Einsatz der Tranche 4 erfolgte schließlich am 15. Dezember 2020. Die Studie endete am 28. Februar 2021.

Tab. 5 zeigt die in den Tranchen realisierten Fälle auf Personenebene und die Rücklaufzahlen. Hierbei wird der Eingang nach Fragebogen, Trockenbluttest^55^ und PCR-Test differenziert. Nur Fälle mit einer gültigen Einverständniserklärung werden als gültige Fälle gezählt. Dies liefert für die Tranchen 1 bis 3 die Rücklaufquoten 50,5 %, 51,4 % und 49,5 %. Lediglich die Tranche 4 weicht mit 20,7 % hiervon deutlich ab. Insgesamt ergibt sich eine Rücklaufquote von 47,7 %. Alle Personen, die einen auswertbaren Trockenblut bzw. PCR-Test abgeliefert haben, erhielten vom RKI Labor nach etwa vier bis fünf Wochen einen persönlichen Ergebnisbericht zu ihren Testergebnissen.

**Table 5:** 

	*Tranche 1*	*Tranche 2*	*Tranche 3*	*Tranche 4*	*Gesamt*
*Bruttostichprobe*					
Haushalte	9072	4499	4410	1593	19.574
Personen	14.535	7181	7078	2881	31.675
*Rücklauf mit gültiger Einverständniserklärung*					
Fragebogen	7329	3687	3498	596	15.110
Trockenblut	7115	3626	3459	581	14.781
PCR	7123	3616	3396	554	14.689

Die Erhebung von Corona-bezogenen Körperflüssigkeiten hat zu einer harten Verweigerung von 4,7 % der angeschriebenen Befragten geführt (vgl. Brix et al. 2021, S. 25). Diese Personen haben gegenüber dem Befragungsinstitut erklärt, dass sie an der Corona-Erhebung nicht teilnehmen wollen und zudem auch künftig nicht mehr durch das SOEP befragt werden wollen. Somit kann an dieser Stelle davon ausgegangen werden, dass es Stichprobenmitglieder gab, die diese über die normale jährliche SOEP-Befragung hinausgehende, zusätzliche und ungewöhnliche Anfrage als belastend oder auch als ungebührlich empfanden. Interessanterweise ist diese Verweigerungsrate vergleichbar mit der überdurchschnittlichen Verweigerung im Jahr 1988 als eine Vermögensbilanz der SOEP-Haushalte mit einem Spezialfragebogen erhoben wurde (vgl. Infratest Sozialforschung 1989, S. 4). Fragen nach dem Vermögen galten als sehr sensitiv und die Sensitivität der Befragten wurde durch den eigenen Vermögens-Fragebogen geradezu verstärkt. Die seit 2002 alle fünf Jahre erhobene Vermögensbilanz der Befragten ist in den normalen SOEP-Personenfragebogen integriert und führt nicht zu überdurchschnittlichen Verweigerungen. Immerhin hat die erhöhte Verweigerung 1988 nicht dauerhaft zu einer niedrigeren Teilnahmebereitschaft geführt (1989 und die folgenden Jahre, vgl. Infratest Sozialforschung 1991, S. 6).

Neben harten Verweigerungen gibt es eine ganze Reihe anderer Gründe für die (temporäre) Nicht-Teilnahme am SOEP. Mit einer effektiven Rücklaufquote^56^ von 47,7 % bei Fragebögen, von 46,7 % bei Trockenbluttests und 46,4 % bei PCR-Tests ist die RKI-SOEP-Spezialerhebung als erfolgreich zu bewerten, vor allem wenn man berücksichtigt, dass die Studie sehr kurzfristig und mit einer Vorankündigung von nur zwei Tagen Vorlauf erfolgte. Die niedrige Response-Rate der Tranche 4 (reine Migrantenstichprobe) ist mit Vorsicht zu interpretieren. Diese Tranche 4 beinhaltet sowohl Personen, die oft umziehen, als auch Personen, die den nur auf Deutsch zur Verfügung gestellten Erhebungs- und Informationsmaterialien vermutlich nicht sehr gut folgen konnten; zumal sie es durch die jährlichen SOEP Befragungen, bei denen zusätzliches Material in vielen Muttersprachen zur Verfügung steht, auch anders gewohnt sind. Das Manko der nur auf Deutsch vorliegenden Materialien war der Kurzfristigkeit der Studienplanung geschuldet. Bei eventuellen weiteren Erhebungen im Rahmen der RKI-SOEP Studie werden die Erhebungsmaterialien in die Hauptsprachen der SOEP-Migrantenstichproben übersetzt werden.

Die Ausschöpfung der RKI-SOEP-Stichprobe liegt deutlich über der Teilnahmebereitschaft der Berlin- und Straubing-Stichproben des RKI (29 bzw. 30 %^57^), aber etwas unter derjenigen der RKI-Hotspot-Studien in Bad Feilnbach und Kupferzell (59 bzw. 63 %^58^) (vgl. Studienprotokoll Santos-Hövener et al. 2020). Die große Motivation derer, die bei RKI-SOEP mitmachten, zeigt sich auch daran, dass nur etwa 3,8 % der eingeschickten Trockenblutproben im Labor nicht analysierbar waren. Und von den 565 Personen, die deswegen um eine Wiederholung der Blutannahme gebeten wurden, haben schließlich 68 % (383 Personen) nochmals Blut eingeschickt (vgl. Brix et al. 2021, S. 22f).

Da die SOEP-Studie eine freiwillige Studie ist und zudem noch ein sensibles Thema umfasst, kann die resultierende Stichprobe von getesteten und befragten Personen sozial selektiv verzerrt sein. Der Vorteil des SOEP sind freilich sehr differenzierte Informationen, auch der lokalen Gegebenheiten, die der Selektivitätsanalyse und der Gewichtung zugrunde gelegt werden können. Ausfallanalysen von Steinhauer et al. (2021) zeigen, dass Personen mit einem schlechten Gesundheitszustand und Personen, die sich Sorgen um die Migration nach Deutschland machen, weniger an der Studie teilnehmen als andere Befragte. Auch der regionale Kontext spielte bei der Teilnahmeentscheidung der SOEP Panellisten eine Rolle. So zeigte sich, dass höhere regionale Inzidenzraten (kumuliert, auf Kreisebene) zum Zeitpunkt der Anfrage um Teilnahme an der Studie eine niedrigere Teilnahmeneigung nach sich zogen. Um die Selektionsprozesse bei statistischen Analysen zu kompensieren, wurde ein komplexes Gewichtungsverfahren durchgeführt, in das etwa 400 Variablen einflossen. Bei diesem Verfahren wurden die verschiedenen Auswahlstufen der Rekrutierung der Stichprobe berücksichtigt. Das heißt, es gab Gewichtungsschritte zur Kontaktierung und zur Teilnahme auf den verschiedenen Ebenen der Befragung, d. h. auf Haushalts- und Personenebene. Die derart abgeleiteten Gewichtungsfaktoren wurden so angepasst, dass aus der Stichprobe abgeleitete, relevante Populationsstatistiken denen der Wohnbevölkerung in Privathaushalten in Deutschland gleichen. Die genauen Selektionsprozesse und Gewichtungsschritte der Studie werden von Steinhauer et al. (2021) beschrieben.

Die gewichteten Ergebnisse zeigen (vgl. SOEP-RKI (2021)), dass bis November 2020 in der Bevölkerung in Privathaushalten die für Testeigenschaften korrigierte Seroprävalenz von IgG-Antikörpern bei 1,3 % lag (das 95 %-Konfidenzintervall beträgt 0,9 bis 1,7 %). Mit zwei Prozent lag die Prävalenz für 18 bis 34 Jährige am höchsten; Geschlechtsunterschiede gab es keine. Weitere Auswertungen zeigen, dass Personen mit niedrigem Bildungsniveau (schulisch und beruflich) im Vergleich zu Hochqualifizierten ein fast doppelt so hohes Infektionsrisiko hatten (vgl. Hoebel et al. 2021b).

Da sich bei etwa einem Drittel der Personen, die im Fragebogen einen positiven Corona-Test vor der Studienteilnahme angaben, keine Antikörper gegen SARS-CoV‑2 nachgewiesen werden konnten, kann man davon ausgehen, dass bis November 2020 1,7 % der Erwachsenen in Privathaushalten in Deutschland eine Infektion mit SARS-CoV‑2 durchgemacht hatten. Daraus ergibt sich unter Berücksichtigung der offiziellen Infektionszahlen, die auf positiven PCR-Tests beruhen, dass es bis November 2020 etwa 1,8-mal (95 % Konfidenzintervall: [1,3 %, 2,5 %]) so viele SARS-CoV‑2-Infizierte gab, wie für diese Zeit an die Gesundheitsämter gemeldet worden waren. Dies impliziert eine deutlich niedrigere Dunkelziffer als sie zu Beginn der Pandemie mit dem Faktor 5 bis 10 vermutet wurde „Dies kann als Zeichen für ein erfolgreiches Zusammenwirken von Teststrategie, Gesundheitswesen und öffentlichem Gesundheitsdienst gewertet werden“, so das Resümee der Studienverantwortlichen (SOEP RKI 2021).

## Ausblick

Der Startpunkt der in diesem Beitrag dargestellten Überlegungen waren die Unsicherheiten bei der Einschätzung des Umfangs der Corona-Epidemie in Deutschland. Dies betraf die Dunkelziffer der Corona Infektionen, ihre räumlichen Konzentrationen sowie die zeitliche Stabilität der räumlichen Cluster. Die Unsicherheiten ergaben sich seit Beginn der Pandemie, da die benutzten Inzidenzzahlen in hohem Grade vom Umfang der Corona-Testungen abhängig sind. Diese Testungen schwankten in Abhängigkeit von der Verfügbarkeit der Tests, den sich ändernden Regelungen zur Testung von Kontaktpersonen und systemrelevanten Personen, aber auch aufgrund von Feiertagen. Wünschenswert wäre eine unabhängige stetige Testung der Bevölkerung im Rahmen einer Studie, die den politischen Entscheidern ein zuverlässiges Bild über den Stand der Pandemie im Lande gibt. Da der Verlauf der Corona Pandemie durch eine hohe Dynamik gekennzeichnet ist, sollte ein solches Messinstrument in zeitlich kurzen Abständen verfügbar sein.

Derartige nationale Teststrategien sind beispielsweise im Vereinigten Königreich oder in Italien installiert worden. Da in Deutschland nur Studien in einzelnen Städten und Landkreisen, mit einer Präferenz für Hotspots durchgeführt wurden, werden in diesem Aufsatz Möglichkeiten diskutiert, wie man anhand laufender Umfragen das Informationsdefizit der offiziellen laufenden Corona-Messungen verringern kann. Hierzu wurden verschiedene Alternativen miteinander verglichen. Im Vorlauf der Arbeiten zu diesem Manuskript wurde nur wenige Monate nach dem Ausbruch der Pandemie vorgeschlagen, das SOEP um eine Corona-Beprobungsstichprobe zu ergänzen (vgl. Rendtel et al. 2020). Dieser Vorschlag wurde vom RKI zusammen mit der SOEP-Gruppe tatsächlich umgesetzt und aus den dabei aufgetretenen Hindernissen kann man lernen, was man besser machen könnte – einige Hinweise gibt der vorliegende Beitrag.

Datenschutz, Ethik und Datenzugang bilden das neuralgische Dreieck dieser neuen Kombination von klassischer Umfrage und medizinischer Beprobung. Hier sind noch viele Fragen und Konfliktfelder offen, die geregelt werden müssen, um sich nicht gegenseitig zu blockieren. Wenn jedoch wie für die RKI-SOEP-Studie brauchbare Regelungen gefunden werden, ermöglicht dieser neue Datentyp weitreichende Analysemöglichkeiten für Sozialmediziner, Ökonomen und Sozialwissenschaftler. Insbesondere die langfristigen Folgen der Corona-Pandemie können auf individueller Ebene in einem Panel aufgedeckt und analysiert werden. Die ökonomischen und sozialen Kosten des Stocherns im Datennebel der Corona-Inzidenzen liegen im Milliardenbereich. Die Statistik-Community sollte klar machen, dass eine genauere statistische Information über pandemische Ereignisse vor allem auch eine höhere Wertschätzung der Statistik als der Wissenschaft von Datenerhebungen und deren Auswertung benötigt.

Eine wichtige Erkenntnis der Genese der RKI-SOEP-Studie für die Bewältigung pandemischer Situationen ist die Notwendigkeit interdisziplinären Arbeitens. Der Vorschlag für eine Kombination eines sozialwissenschaftlichen Panels mit einer medizinischen Beprobung wurde von Forschenden aus ganz unterschiedlichen Bereichen und Fächern entwickelt: Epidemiologie, Umfrageforschung, medizinische Biometrie, Pharmakologie, Sozial‑, Verhaltens- und Wirtschaftswissenschaften. Unter den Unterstützern trafen sich aber auch unterschiedliche Forschungsinstitutionen: staatliche Forschungsinstitute, unabhängige Forschungsinstitute, Universitäten und Unternehmen, vgl. die Projektskizze der RKI-SOEP Studie bei Rendtel et al. (2020).

Als Ausblick sei darauf hingewiesen, dass man von den Epidemiologen lernen kann, dass aussagekräftige Modelle auch mit weitaus geringeren Fallzahlen schätzbar sind, als es Survey-Statistiker mit ihrem Design-basierten Ansatz gewohnt sind. Allerdings gehören diese Multi-State-Modelle nicht zum Standardinventar der Survey-Statistik.

**Figure 8:**
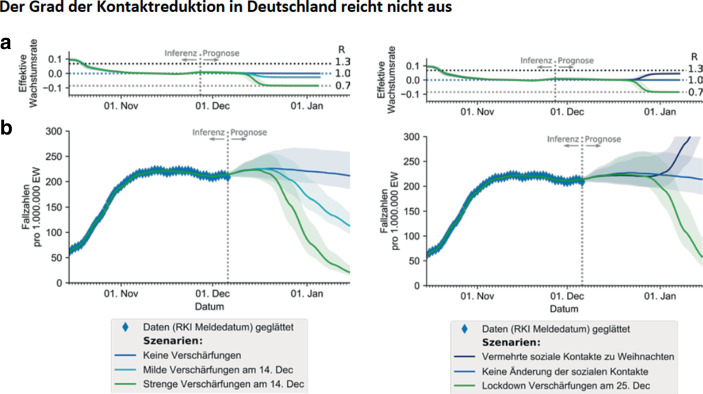

Modellbildungen der mathematischen Epidemiologie hatten in der Anfangsphase der Corona Pandemie durchaus ihre Berechtigung, weil einfach noch keine empirischen Erfahrungen mit dem Verlauf dieser neuartigen Erkrankung vorlagen. So gestattet das SIR Modell^59^ Prognosen für das Auftreten weiterer Infektionen und deren Abschwellen im Laufe der Zeit. Doch spätestens seit der Kenntnis von regional völlig unterschiedlichen Infektionsclustern war die Annahme eines einheitlichen einfachen Ansteckungsmodells für ganz Deutschland nicht mehr realitätsgerecht und führte zu unrealistischen Prognosen von Infektionszahlen. Beispielhaft sei hier aus der Ad-hoc Stellungnahme der Nationalen Wissenschaftsakademie Leopoldina vom 8. Dezember 2020 „Die Feiertage und den Jahreswechsel für einen harten Lockdown nutzen“ zitiert^60^. Dort wird auf S. 4 mit der folgenden Abbildung (hier Abb. 8) für strenge Verschärfungen der Kontaktregelungen argumentiert. Die Annahme, man könne das Kontaktgeschehen über die Weihnachtsfeiertage über einen einheitlichen, für ganz Deutschland gültigen R‑Wert modellieren und steuern, erscheint wirklichkeitsfremd. Abb. 2 zeigt auf Basis der Kreisdaten des RKI ein ganz anderes Bild von Deutschland zum Zeitpunkt der Leopoldina-Prognose: Ein hochgradig diverses regionales Infektionsgeschehen. Und schließlich liegen die Prognosen für den tatsächlich beschlossenen harten Lockdown meilenweit neben der tatsächlichen Entwicklung der Infektionszahlen. Abb. 5 zeigt trotz der beschlossenen Verschärfung des Lockdowns vor den Weihnachtstagen einen scharfen Anstieg der Inzidenzzahlen, die nach der Prognose doch hätten fallen müssen. Auch die Wiedererreichung von Inzidenzzahlen unter 50 war für den Jahreswechsel 2020/2021 vorhergesagt worden. Eingetreten ist dieses Niveau aber erst Mitte Februar 2021.

Es lohnt sich, die oben aufgeworfenen Fragestellungen auch nach der Eindämmung der jetzigen Corona-Pandemie zu klären, denn vermutlich dürfte diese Pandemie nicht die letzte gewesen sein. Daneben wären auch belastbare kommunikationswissenschaftliche Analysen wichtig, um die Krisen-Kommunikation der Verantwortlichen zu verbessern und damit die Pandemie besser zu beherrschen. Es spricht jedenfalls nicht für die Kommunikationsstrategie der Bundes- und Länderregierungen, wenn ein erkennbar unzuverlässiger Indikator wie die gemessene Inzidenz der Infektionen im Mittelpunkt der Kommunikation steht und am Ende gar in ein Bundesgesetz als zentraler Indikator einfließt.

Der vollständige wissenschaftliche und gesellschaftliche Nutzen der RKI-SOEP-Studie wird sich erst mittel- und langfristig zeigen, wenn mit Hilfe der Längsschnittdaten der Verlauf der Pandemie besser verstanden werden kann und die langfristigen gesundheitlichen und sozialen Folgen einer Infektion analysiert werden können. Zu diesem Zweck wird – wiederum mit Förderung durch das BMG – ab Oktober 2021 der Antikörperstatus^61^ zusammen mit einem längeren Fragebogen mit dem SOEP erhoben.

## Footnotes

1 Mittlerweile wurden die Gesundheitsämter zumindest teilweise technologisch und personell nachgerüstet, so dass die Begrenzung durch den Grenzwert von 50 nicht mehr als gegeben betrachtet wird. Zur Unterstützung der Gesundheitsämter bei der Kontaktverfolgung wird auf eine spezielle Software gesetzt, siehe https://www.sormas-oegd.de/ (Abruf am 16.07.2021).

2 Nach Selbsteinschätzung der Gesundheitsämter war deren Fähigkeit zur Nachverfolgung von Kontaktpersonen auch zu Hochzeiten der zweiten Corona-Welle noch vorhanden, vgl. https://www.tagesspiegel.de/politik/willkuer-bei-der-inzidenz-fast-alle-gesundheitsaemter-koennen-mit-werten-ueber-50-arbeiten/26923572.html (Abruf am 16.07.2021).

3 Die Ermittlung der Personen, die eventuell eine Ansteckung verursacht haben könnten, ist im Alltag schwierig. Wer mit dem Öffentlichen Nahverkehr zur Arbeit fährt und am Arbeitsplatz mit Publikumsverkehr arbeitet, wird es schwer haben, alle Kontaktpersonen zu nennen. Nach Menne (2021) können die Gesundheitsämter nur noch in einem von 6 Fällen ermitteln, wo sich die Infizierten angesteckt haben.

4 Die Abbildungen geben geschätzte lokale Inzidenzen wieder. Diese Karten werden im Abschnitt zur räumlichen Intensität der Corona Pandemie näher erläutert. Die Daten für die Infektionsfälle pro Landkreis folgen den Angaben des RKI, vgl. https://www.rki.de/DE/Content/InfAZ/N/Neuartiges_Coronavirus/Daten/Fallzahlen_Kum_Tab.html (Abruf am 16.07.2021).

5 Vgl. https://web.de/magazine/panorama/rostock-kampf-coronavirus-35509126 (Abruf 19.07.2021).

6 https://www.deutschlandfunkkultur.de/oberbuergermeister-claus-ruhe-madsen-erfolgreiches.1008.de.html?dram:article_id=489976 (Abruf am 16.07.2021).

7 Auch hier wurden zunächst nur die Personen mit Krankheitssymptomen getestet. Außerdem änderte sich die Definition der reinen Kontaktperson über den Pandemieverlauf hinweg zu Kontaktpersonen 1. und 2. Ordnung, von denen nur Kontaktpersonen 1. Ordnung getestet wurden.

8 Vgl. RKI-Tagesbericht vom 20.04.2021 Abb. 7 unter https://www.rki.de/DE/Content/InfAZ/N/Neuartiges_Coronavirus/Situationsberichte/Apr_2021/2021-04-20-de.pdf (Abruf am 16.07.2021).

9 Erst mit dem Inkrafttreten der sogenannten Bundesnotbremse am 24.04.2021 in Kalenderwoche 16 wurde der Präsensunterricht in Landkreisen mit einem Inzidenzwert über 165 wieder eingestellt.

10 https://www.focus.de/gesundheit/coronavirus/inzidenz-bei-kindern-teils-ueber-500-warum-sie-ploetzlich-haupttreiber-der-pandemie-sind_id_13214105.html (Abruf am 16.07.2021).

11 „The seroepidemiological investigation for COVID‑19 virus infection is a population-based, age-stratified prospective study“ (S. 5): (WHO), Population-based age-stratified seroepidemiological investigation protocol for COVID‑19 virus infection, Version 1.1, March 17 2020: https://apps.who.int/iris/bitstream/handle/10665/331656/WHO-2019-nCoV-Seroepidemiology-2020.1-eng.pdf (Abruf 19.07.2021).

12 Diese Studie wird nur für England durchgeführt. Die anderen Teile des Vereinigten Königreichs führen separate Studien durch.

13 https://www.ndm.ox.ac.uk/files/coronavirus/covid-19-infection-survey/protocolcovid19infectionsurveyv8-120210412.pdf, für Ergebnisse vgl. https://www.ons.gov.uk/peoplepopulationandcommunity/healthandsocialcare/conditionsanddiseases/bulletins/coronaviruscovid19infectionsurveypilot/previousReleases (Abruf am 16.07.2021).

14 https://www.thelancet.com/journals/lanpub/article/PIIS2468-2667(20)30256-5/fulltext (Abruf am 16.07.2021).

15 Erst ab Sommer 2021 weist das RKI eine Hospitalisierungsinzidenz aus, die die Anzahl der Corona-induzierten Krankenhauseinweisungen in 7 Tagen pro 100 Tausend Einwohnern misst. Allerdings benutzt das RKI als Stichtag nicht den Tag der Krankenhauseinweisung, sondern den Tag des Infektionsnachweises. Da die Krankenhauseinweisungen nur mit Verzögerungen nach der Infektion auftreten, führt dies zu einer Unterschätzung der Anzahl der Krankenhauseinweisungen, siehe auch Weigert et al. (2021). Weiterhin weist das RKI die Hospitalisierungsinzidenz nur auf einem überregionalen Level (Bundesland) aus. Bei regional stark schwankenden Infektionszahlen sind derartige überregionale Inzidenzwerte nicht geeignet, konkrete Überlastungen des Gesundheitssystems anzuzeigen.

16 Dieser Test wird auch als Test auf Seropositivität bezeichnet.

17 https://www.rki.de/DE/Content/Gesundheitsmonitoring/Studien/cml-studie/Factsheet_Berlin-Mitte.html (Abruf am 16.07.2021).

18 Einen Überblick findet man unter https://www.rki.de/DE/Content/InfAZ/N/Neuartiges_Coronavirus/AK-Studien/AKS_Allgemein.html (Abruf am 16.07.2021).

19 Das Studienprotokoll der Corona-Monitoring lokal Untersuchung findet man unter https://www.rki.de/DE/Content/Gesundheitsmonitoring/Gesundheitsberichterstattung/GBEDownloadsJ/JoHM_S5_2020_Studienprotokoll_CORONA_MONITORING_lokal.pdf?__blob=publicationFile (Abruf am 16.07.2021).

20 Das geschilderte Mess-Problem im Hinblick auf eine aktuelle Schätzung der Dunkelziffer birgt zugleich eine relevante eigenständige Forschungsfrage. Auf die Möglichkeiten von relevanten Längsschnittuntersuchungen des Antikörper- bzw. Immun-Status mit Hilfe einer repräsentativen Bevölkerungserhebung soll an dieser Stelle aber nicht eingegangen werden.

21 Diese Studie war von vorneherein nur für zwei Zeitpunkte ausgelegt. Während der ersten Messung gab es wegen der geringen Corona-Inzidenz deutliche Fallzahlprobleme, d. h. sehr ungenau geschätzte Inzidenzen. Zum zweiten Zeitpunkt mitten im Aufschwung der zweiten Corona-Welle hatte man die Stichprobengröße aus nicht dokumentierten Gründen halbiert.

22 Die Testung von Kindern scheint auch in dieser Studie ein Problem gewesen sein. Dieses Problem zeigt sich auch bei der unten beschriebenen RKI-SOEP Studie, wo ebenfalls nur Jugendliche ab dem Alter von 18 Jahren getestet werden durften. Angesichts der seit dem Frühjahr 2021 massenhaft eingesetzten Testung von Kindern in Schulen und Kitas ist diese Restriktion kaum nachvollziehbar. Insbesondere lassen die Ergebnisse dieser Studie vermuten, dass gerade in dieser Gruppe die Dunkelziffer besonders hoch ist. Umgekehrt bedeutet dies, dass die ausgewiesene Dunkelziffer den Wert in der Gesamtpopulation unterschätzt.

23 https://interaktiv.tagesspiegel.de/lab/corona-analyse-in-welchen-regionen-die-zahlen-wieder-steigen/ (Abruf am 16.07.2021).

24 https://www.inwt-statistics.de/blog-artikel-lesen/COVID-19_Karte_der_lokalen_7-Tage-Inzidenz_im_Zeitverlauf.html (Abruf am 16.07.2021).

25 http://www.salute.gov.it/portale/news/p3_2_1_1_1.jsp?lingua=italiano&menu=notizie&p=dalministero&id=5012 (Abruf am 16.07.2021).

26 https://www.rki.de/SharedDocs/Bilder/InfAZ/neuartiges_Coronavirus/Teststrategie.jpg;jsessionid=7110EB4B96C8933846944336F2A254D6.internet072?__blob=poster&v=11 (Abruf am 16.07.2021).

27 https://www.deutschlandfunk.de/coronaviren-im-abwasser-messungen-sind-den-infektionszahlen.676.de.html?dram:article_id=496121 (Abruf am 16.07.2021).

28 Auf das Problem einer möglichen Verzerrung der Ergebnisse durch Nonresponse wird in einem separaten Anschnitt weiter unten eingegangen.

29 http://www.klinikum.uni-muenchen.de/Abteilung-fuer-Infektions-und-Tropenmedizin/de/COVID-19/KoCo19/Aktuelles/index.html (Abruf am 16.07.2021).

30 Im MZ wird die Stichprobe gleichmäßig auf die 12 Monate verteilt, um die Saisonalität des Arbeitsmarkts zu erfassen.

31 Vgl. die Diskussion im Tagesspiegelforum unter https://interaktiv.tagesspiegel.de/lab/hotspots-und-rechte-haengen-afd-hochburgen-und-corona-hotspots-zusammen/ (Abruf am 16.07.2021).

32 PASS steht für das Panel Arbeitsmarkt und Soziale Sicherung, das von der Bundesagentur für Arbeit betrieben wird, siehe https://fdz.iab.de/de/FDZ_Individual_Data/PASS.aspx (Abruf am 16.07.2021).

33 SHARE steht für den Survey on Health, Aging and Retirement in Europe, http://www.share-project.org/home0.html (Abruf am 16.07.2021).

34 Die SOEP-COV Umfrage wurde als telefonische Umfrage bereits im April 2020 während des ersten Lockdowns durchgeführt.

35 Die Auswahl dieser Stichprobe wird im Abschlussbericht nicht näher beschrieben. Es ist nicht klar, ob es sich hierbei um eine Haushaltsstichprobe oder eine Personenstichprobe handelt. Allerdings werden nur erwachsene Personen in die Stichprobe aufgenommen. Es wird auch nicht klar wie die 11 Tsd. Probanden aus den insgesamt 30 Tsd. Stichprobenmitgliedern ausgewählt wurden. Es könnte sich auch hierbei um unkontrollierten Nonresponse handeln. Forsa kalibriert die Population der Probanden nach Alter, Geschlecht und Region.

36 Das Konzept der Erweiterung einer Basiserhebung um medizinische Tests wurde mit dem Sozio-oekonomischen Panel (SOEP) ab September 2020 als RKI-SOEP-Stichprobe realisiert. Die Überlegungen zur Umsetzung dieses Konzepts werden in Abschn. 5 unten dargestellt.

37 Zu den Unterschieden im Stichprobendesign von NAKO-Gesundheitsstudie und einer geographisch repräsentativen Studie siehe https://www.ratswd.de/dl/RatSWD_WP_237.pdf. Zugegriffen: 16. Juli 2021.

38 Bei der NAKO-Gesundheitsstudie kommt die Altersbegrenzung dazu (aktuell sind Personen im Alter von 73plus nicht in der Stichprobe).

39 EU-SILC steht für EU Statistics on Income and Living Conditions, vgl. https://www.destatis.de/DE/Themen/Gesellschaft-Umwelt/Einkommen-Konsum-Lebensbedingungen/Lebensbedingungen-Armutsgefaehrdung/Methoden/EU-SILC.html (Abruf am 16.07.2021).

40 In der Diskussion des Artikels von Schnell und Smid (2020) wird explizit der hohe Zeitaufwand für die Erstellung einer Stichprobe durch die Einwohnermeldeämter von mehr als 6 Monaten beklagt.

41 Dies ist durch das Infektionsschutzgesetz vorgeschrieben.

42 https://www.ifsg-online.de/antrag-taetigkeitsverbot.html (Abruf am 16.07.2021).

43 https://www.berlin.de/sen/gesundheit/service/gesundheitsberichterstattung/veroeffentlichungen/kurz-informiert/#Corona20 (Abruf 19.07.2021).

44 Der Test zeigt nur eine akute Virus-Infektion an, und zwar über 7 bis 14 Tage nach dem Einsetzen der Symptome. Sobald eine Immunreaktion einsetzt, verringert sich die Viruslast und die Sensitivität des Tests sinkt.

45 Allerdings durften aufgrund einer Entscheidung des Datenschutzbeauftragten des RKI nur Personen über 18 Jahre getestet werden. Dies bedeutet, dass praktisch keine Aussagen über die Dunkelziffer bei Jugendlichen aus der Stichprobe gewonnen werden können. Diese Entscheidung steht im Gegensatz zum Vorgehen in der COALA-Studie, die ebenfalls vom RKI geleitet wird, vgl. https://www.rki.de/DE/Content/InfAZ/N/Neuartiges_Coronavirus/Projekte_RKI/KiTa-Studie-Berichte/KiTAStudie_03_2021.pdf (Abruf am 16.07.2021). Dort werden Anlass-bezogen Kinder aus Kitas mit Infektionsfällen zu Hause von ihren Eltern getestet. Empfohlen wird die Testung 14 Tage lang täglich zu wiederholen.

46 Allerdings ist hier die Dunkelziffer vermutlich deutlich geringer als bei der übrigen Bevölkerung, vgl. Wagner et al. (2021).

47 Diese Möglichkeit konnte allerdings durch die Intervention eines der beteiligten Datenschutzbeauftragten nicht realisiert werden.

48 „serial sampling“ in der Terminologie der WHO.

49 WHO, a. a. O., S. 5f.

50 Das Infektionsgeschehen in der ersten und zweiten Welle unterscheidet sich deutlich. Während in der ersten Welle das SARS-Cov2 Virus neu in die Population eingetragen wurde, war es in der zweiten Welle in der Population schon weit verbreitet.

51 Eine zweite Testung ist im Budget der RKI-SOEP Studie nicht vorgesehen.

52 Die 2019er-Erhebung ist bislang nicht öffentlich dokumentiert. Es liegen detaillierte Unterlagen und entsprechende Probandenaufklärungen vor, die neben den Datenschützern des DIW Berlin und des Erhebungsinstituts KANTAR von der zentralen Ethikkommission der Max-Planck-Gesellschaft geprüft wurden.

53 Die Kosten der RKI-SOEP Studie liegen mit insgesamt 2,7 Mio. € Erhebungskosten gemessen an sozialwissenschaftlichen Erhebungen hoch, aber gemessen an gesundheitswissenschaftlichen Erhebungen in einem niedrigen Bereich. Bei einer Bruttostichprobe von insgesamt 31.000 befragten Personen, ergeben sich Kosten von ungefähr 90 € pro Befragtem. Auch wenn man die RKI-SOEP-Studie mit anderen Corona-Projekten des Bundesgesundheitsministeriums vergleicht, sind die Kosten überschaubar. So beliefern sich die Kosten für die Corona-Warn-App auf geschätzte 67 Mio. € (https://www.bundesregierung.de/breg-de/themen/corona-warn-app (Abruf 19.07.2021)). Das ist das 25-fache der Feldkosten der RKI-SOEP-Studie! Der Nutzen dieser App erwies sich im Nachherein als sehr beschränkt (vgl. Z. B. Dehmel et al. (2020), https://www.spiegel.de/netzwelt/netzpolitik/corona-warn-app-warum-viele-sie-nutzen-und-viele-nicht-a-f7956afd-6cd8-4d8f-9835-fd40fe0fe022 (Abruf am 16.07.2021)).

54 Die beiden reinen Migrationsstichproben M1 und M2 entstammen einem Kooperationsprojekt mit dem Institut für Arbeitsmarktforschung (IAB). Die Klärung aller datenschutzrechtlichen Aspekte dauerte durch die Einbindung eines zusätzlichen Kooperationspartners (neben dem RKI) zusätzliche vier Wochen. M1 und M2 sind andere Stichproben als die oben angesprochenen und ausgeschlossenen Geflüchteten-Stichproben.

55 Im Rahmen der Studie erwiesen sich im Labor die Trockenbluttest von 383 Fällen als nicht auswertbar. Den betroffenen Personen wurde die Möglichkeit eingeräumt, die Tests zu wiederholen.

56 Response Rate 6 nach AAPOR Code für alle Fälle mit gültiger Einverständniserklärung, siehe https://www.aapor.org/Standards-Ethics/Standard-Definitions-(1).aspx (Abruf am 16.07.2021).

57 https://www.rki.de/DE/Content/Gesundheitsmonitoring/Studien/cml-studie/Factsheet_Berlin-Mitte.html und https://www.rki.de/DE/Content/Gesundheitsmonitoring/Studien/cml-studie/Factsheet_Straubing.html (Abruf am 16.07.2021).

58 https://www.rki.de/DE/Content/Gesundheitsmonitoring/Studien/cml-studie/Dokumente/Factsheet_Bad%20Feilnbach.pdf?__blob=publicationFile und https://www.rki.de/DE/Content/Gesundheitsmonitoring/Studien/cml-studie/Factsheet_Kupferzell.html (Abruf am 16.07.2021).

59 SIR steht für susceptible (ansteckungsgefährdet), infected, removed (aus Ansteckungsgeschehen), vgl. https://de.wikipedia.org/wiki/SIR-Modell (Abruf am 16.07.2021).

60 https://www.leopoldina.org/publikationen/detailansicht/publication/coronavirus-pandemie-die-feiertage-und-den-jahreswechsel-fuer-einen-harten-lockdown-nutzen-2020/ (Abruf am 16.07.2021).

61 Den akuten Infektionsstatus (mit einem PCR-Test festgestellt) zu erheben birgt die Gefahr, dass Stichproben-Mitglieder nicht mitmachen, da sie im Falle einer Infektion in Quarantäne gehen müssen. Der Verzicht auf einen PCR-Test für die Studie könnte die Teilnahmebereitschaft erhöhen und für die Bekämpfung der Pandemie wiegt der Verzicht nicht schwer, da die Zahl in akut Infizierten in der Stichprobe für das Pandemiegeschehen nur eine marginale Rolle spielt.
